# Repositioning of anti-dengue compounds against SARS-CoV-2 as viral polyprotein processing inhibitor

**DOI:** 10.1371/journal.pone.0277328

**Published:** 2022-11-16

**Authors:** Leena H. Bajrai, Arwa A. Faizo, Areej A. Alkhaldy, Vivek Dhar Dwivedi, Esam I. Azhar

**Affiliations:** 1 Special Infectious Agents Unit – BSL3, King Fahd Medical Research Center, King Abdulaziz University, Jeddah, Saudi Arabia; 2 Biochemistry Department, Faculty of Sciences, King Abdulaziz University, Jeddah, Saudi Arabia; 3 Department of Medical Laboratory Sciences, Faculty of Applied Medical Sciences, King Abdulaziz University, Jeddah, Saudi Arabia; 4 Clinical Nutrition Department, Faculty of Applied Medical Sciences, King Abdulaziz University, Jeddah, Saudi Arabia; 5 Center for Bioinformatics, Computational and Systems Biology, Pathfinder Research and Training Foundation, Greater Noida, India; 6 Bioinformatics Research Division, Quanta Calculus, Greater Noida, India; Alagappa University, INDIA

## Abstract

A therapy for COVID-19 (Coronavirus Disease 19) caused by Severe Acute Respiratory Syndrome Coronavirus 2 (SARS-CoV-2) remains elusive due to the lack of an effective antiviral therapeutic molecule. The SARS-CoV-2 main protease (M^pro^), which plays a vital role in the viral life cycle, is one of the most studied and validated drug targets. In Several prior studies, numerous possible chemical entities were proposed as potential M^pro^ inhibitors; however, most failed at various stages of drug discovery. Repositioning of existing antiviral compounds accelerates the discovery and development of potent therapeutic molecules. Hence, this study examines the applicability of anti-dengue compounds against the substrate binding site of M^pro^ for disrupting its polyprotein processing mechanism. An *in-silico* structure-based virtual screening approach is applied to screen 330 experimentally validated anti-dengue compounds to determine their affinity to the substrate binding site of M^pro^. This study identified the top five compounds (CHEMBL1940602, CHEMBL2036486, CHEMBL3628485, CHEMBL200972, CHEMBL2036488) that showed a high affinity to M^pro^ with a docking score > -10.0 kcal/mol. The best-docked pose of these compounds with M^pro^ was subjected to 100 ns molecular dynamic (MD) simulation followed by MM/GBSA binding energy. This showed the maximum stability and comparable ΔG binding energy against the reference compound (X77 inhibitor). Overall, we repurposed the reported anti-dengue compounds against SARS-CoV-2-M^pro^ to impede its polyprotein processing for inhibiting SARS-CoV-2 infection.

## 1. Introduction

Pharmaceutical research into the discovery and development of therapeutic compounds has expanded rapidly since the emergence of COVID-19 (a disease caused by SARS-CoV-2 infection) [1–3]. Target-based drug discovery has been investigated exponentially due to the release of experimental structures of essential structural and non-structural proteins of SARS-CoV-2 [4–6]. These proteins are used as drug targets in computational structure-based drug design, where virtual screening is performed against these targets to find the most potent antagonist compound [7–9]. The protease enzyme is involved in viral protein maturation by breaking down polypeptide sequences, which concludes the infection life cycle of the virus [10, 11], making the protease enzyme a promising therapeutic target [12, 13]. Main protease (M^pro^)^’^ functional role in SARS-CoV-2 growth established its potential clinical use in the drug discovery pipeline [14, 15]. In the case of other viruses, several therapeutic molecules have been developed to target their corresponding M^pro^ structure. Several compounds, including nelfinavir, ritonavir, indinavir, atazanavir, saquinavir, lopinavir, amprenavir, darunavir, and tipranavir, are effective protease inhibitors in HIV [16]. Similarly, among hepatitis C virus compounds, sofobuvir, voxilaprevir, glecaprevir, grazoprevir, paritaprevir, asunaprevir, ritonavir, telaprevir, and boceprevir were the FDA approved therapeutic molecules [17].

The M^pro^ protein of SARS-CoV-2 is a cysteine protease that cleaves itself between the nsp4 and nsp6, followed by the cleavage of other polypeptide regions resulting in the maturation of viral proteins, which is required for virus replication [18–20]. M^pro^ is a homodimer protein; each protomer contains three domains, viz. Domain-I from 8 to 101, Domain II from 102 to 184, and Domain III from 201 to 303 amino acid residues. Domains I and II contain antiparallel β-barrel structures and together, they form a cleft possessing a Cys-His catalytic dyad which serves as a substrate binding site similar to other coronavirus families. However, Domain III is a helical domain with 5 α-helices that form a globular structure and is connected to Domain II via a 15-residue long loop structure. [21–23]. The N-terminal finger (residues 1 to 7) located between domain II and domain III of each protomer forms a connecting link between the protomer forming the substrate binding site in a cleft which is located between domain I and domain II [24–28]. The monomeric SARS-CoV-2 M^pro^ is functionally inactive for polyprotein processing and therefore the dimerization interface serving the substrate binding site is targeted to develop potential therapeutics in this study [29, 30]. Multiple crystal structures of SARS-CoV-2 M^pro^ have been solved, which facilitated novel drug design and drug repurposing [31–47].

Structure-based virtual screening is widely used in drug discovery using the SARS-CoV-2 M^pro^ protein crystal structure, and the ultra large chemical library was screened against the active site where the compound selection was guided by the crystal structure information and docking-based screening [48]. Ligand-based drug design (LBDD) was also used in several studies where pharmacophores were designed using known inhibitors and further screened in the ZINC library [49, 50]. A study illustrated computational tools and techniques to design peptidomimetic inhibitors against SARS-CoV-2 M^pro^ [51]. The natural compound library was screened in several cases to identify the potential M^pro^ inhibitor using molecular docking and MM/PBSA method [52]. The efficiency of current antiviral compounds against SARS-CoV-2 M^pro^ was also evaluated using molecular docking and modelling as part of a drug repurposing effort [53].

Moreover, experimental findings of inhibitors were also validated using molecular dynamics simulation to understand the molecular level interaction between proteins and small molecules [54]. Among natural compounds or their derivatives, flavonoid compounds were extensively used in various computation screening studies against M^pro^ [55–62]. Many antiviral compounds were evaluated computationally against SARS CoV-2 M^pro^ to detect their repurposing effect. However, experimentally validated anti-dengue compounds were not comprehensively explored against SARS-CoV-2 M^pro^. In recent work, Murtuja et al. demonstrated the development of DENV inhibitors with a targeted comparison of a protease between DENV and SARS-CoV-2 [62].

In this study, a structure-based screening was performed against the SARS-CoV-2 M^pro^ using the known DENV inhibitors. These are experimentally validated DENV inhibitors (antiviral compounds). Therefore with high hopes of finding promising antiviral therapeutic molecules against another viral protein, i.e., SARS-CoV-2 M^pro^, they were tested by employing docking and molecular dynamics simulation techniques. In the first phase, the binding of these compounds with M^pro^ at its active site was determined using multilevel docking with standard and additional precision algorithms of the Glide tool. Top 5 compounds selected based on docking scores were: **(1)** N-benzoyl-L-norleucyl-L-lysyl-L-arginyl-2-phenyl-L-glycinal, (CHEMBL1940602) **(2)** (E)-7-[8-[(E)-6-carboxy-1-phenylhex-1-en-3-yl]-5,7-dihydroxy-4-oxo-2-phenyl-2,3-dihydrochromen-6-yl]-7-phenylhept-5-enoic acid (CHEMBL2036486), **(3)** [2-[(2S,3R,4S,5S,6R)-3-benzoyloxy-6-(benzoyloxymethyl)-4,5-dihydroxyoxan-2-yl]oxy-5-hydroxyphenyl]methyl (2R,3S,4S)-1,2,3,4-tetrahydroxy-5-oxocyclohexane-1-carboxylate (Flacourtoside D) (CHEMBL3628485), **(4)** [2-[(2S,3R,4S,5S,6R)-3-benzoyloxy-6-(benzoyloxymethyl)-4,5-dihydroxyoxan-2-yl]oxy-5-hydroxyphenyl]methyl (1R,2R,6R)-6-benzoyloxy-1,2-dihydroxy-5-oxocyclohex-3-ene-1-carboxylate (Flacourtoside F) (CHEMBL200972), and **(5)** (2S)-6-amino-N-[(1S)-2-amino-2-oxo-1-phenylethyl]-2-[[(2S)-3-[4-(diaminomethylideneamino)phenyl]-2-(3 phenylpropanoylamino) propanoyl] amino]hexanamide (CHEMBL2036488). In addition, this study used a known M^pro^ inhibitor to compare the binding of screened compounds against the protein target. The control compound (X77 inhibitor: N-(4-tert-butylphenyl)-N-[(1R)-2-(cyclohexylamino)-2-oxo-1-(pyridin-3-yl) ethyl]-1H-imidazole-4-carboxamide) was sourced from the crystal structure of protein where it was co-crystallized with SARS-CoV-2 M^pro^ as a potential non-covalent inhibitor. MM/GBSA binding energy of all these top 5 screened compounds and the control (X77) was calculated over the complete trajectory of 100 ns MD simulation time. Overall, this study demonstrates the potential of five known DENV inhibitors to suppress SARS-CoV-2-M^pro^ activity.

## 2. Materials and methods

This study involved comprehensive molecular docking and molecular dynamic simulation in screening anti-DENV compounds against SARS-CoV-2 M^pro^.

### 2.1 Structures collection

**Fig 1:**
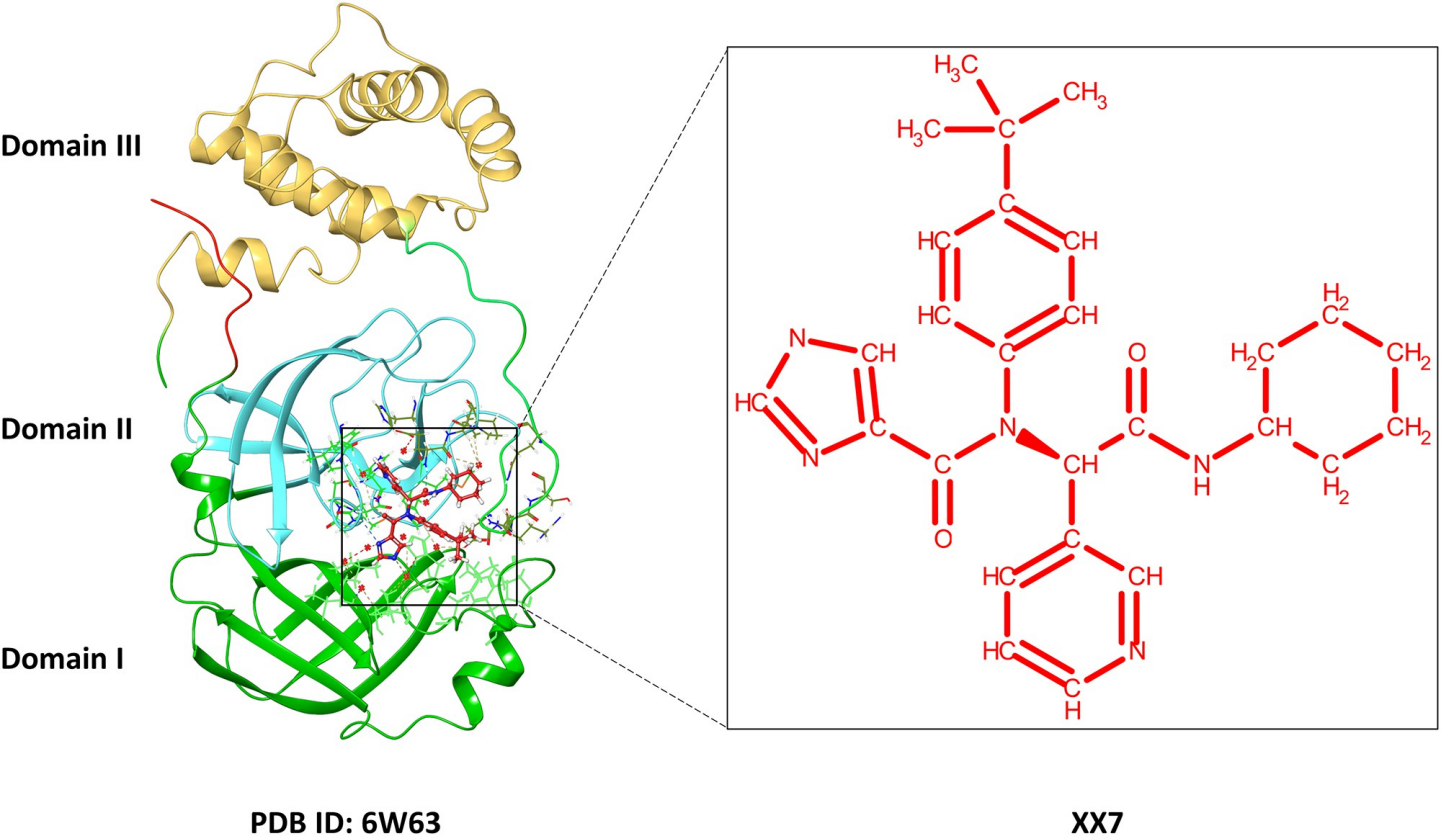
Crystal structure of M^pro^ (PDB ID: 6W63) representing Domain I, II and III with the co-crystalized ligand (PDB code: X77).

The protein structure of SARS-CoV-2 M^pro^ was sourced from the protein data bank (PDB) [63] with PDB ID: 6W63 (https://www.rcsb.org/structure/6W63). This structure contains the SARS-CoV-2 main protease solved using the X-ray diffraction technique at 2.10 Å resolution. It consists of single chain ‘A’ with 306 amino acids with a broad spectrum non-covalent in inhibitor N-(4-tert-butylphenyl)-N-[(1R)-2-(cyclohexylamino)-2-oxo-1-(yridine-3-yl)ethyl]-1H-imidazole-4-carboxamide labelled as X77 in the PDB entry. As illustrated in Fig 1, this co-crystallized inhibitor is placed at the interface of domains I and II of the protein.

The compound library was prepared from DenvInD databases (https://webs.iiitd.edu.in/raghava/denvind/) that store the active inhibitors against the dengue virus [64]. The study started with 330 compounds collected from the DenvInD database that were further prepared using the default parameters of the LigPrep panel in the Schrödinger suite [65]. During the ligand preparation, pH was set at 7.0 ± 2.0, and the OPLS-2005 force field was used to assign charge and radius to each ligand atom.

### 2.2 Docking preparation

This study primarily depends on the structure-based virtual screening (SBVS), where the receptor structure (PDB ID: 6W63) was prepared for docking using the protein preparation wizard of the Schrödinger suite [66]. Here, the missing atoms were added to the protein structure with the assignment of partial and formal charges to the molecule. Co-crystalized water molecules were removed to allow free docking of new compounds to the protein’s active site.

The information from a native (X77 inhibitor) ligand bound with the structure was used to create a docking grid that covered all essential residues grid centre at -20.46 (X), 18.11 (Y), -26.9 Å (Z) region. The inner-box size 10 X10 X 10 Å and outer-box size 25.094 X 25.094 X 25.094 were set along X, Y, and Z axes using the grid generation tool of the Schrödinger suite to allow the ligand docking with the SARS-CoV-2 M^pro^ active site with all possible confirmations. His41 and Cys145 constitute the catalytic duo of SARS-CoV-2 M^pro^, and the grid box was constructed around these two catalytic residues. In addition, residues that demonstrated active contact with X77 inhibitor in its PDB structure were considered during grid generation. Glu166, His163, and Gly143 form direct H-bonds with X77. Other polar and nonpolar residues close to the ligand also assisted in making the grid. These included Gln189, Asp187, His164, Thr25, Thr26, Asn142, Met165, Phe140, Ser144, and Leu141 residues.

### 2.3 Structure-based virtual screening workflow

In the first phase, compounds were screened using high throughput virtual screening of the Schrödinger suite. Later the filtered compounds were docked in the defined grid using the Glide standard precision (SP) and extra precision (XP) modules of the Schrödinger suite [67–70]. Reference compound X77 was removed from the PDB structure and redocked at the defined grid site. All docked poses were analysed for intermolecular interactions using the default settings of Maestro v12.9, and 3D and 2D interaction diagrams were generated using the free academic edition of Maestro v12.9 [71].

### 2.4 Molecular dynamic simulation

The top poses of five compounds and the reference compound were simulated further using the Desmond-maestro 2020–4 academic package [72, 73]. Every docked pose was placed in a 10 Å × 10 Å × 10 Å orthorhombic box and an explicit solvent molecule (TIP4P: transferable intermolecular potential 4 point) was added with 0.15 M salt concentration to bring the system to a physiological state. Later, the system was neutralized by adding the appropriate number of Na^-^ and Cl^-^ ions. In the first phase of simulation, the complete system was minimized under the default parameters of the simulation. Post minimization, a 100 ns simulation was performed under the OPLS-2005 force field at 300 K temperature. The simulation trajectory was further analysed using the root mean square deviation (RMSD) of Cα atoms of protein using Eq (1).

$$RMSD_{X}=\sqrt{\frac{1}{N}}\sum_{i=1}^{N}{\left(r_{i}^{\prime }\left(t_{x}\right)-\;r_{i}\left(t_{ref}\right)\right)}^{2}$$ (1)

***N***: the number of atoms selected; ***t***_***ref***_: reference time at zero interval; ***r***_***i***_: position of the atoms under evaluation in frame x; $r_{i}^{'}$: position of the atoms in the reference frame; ***t***_***x***_: time frame for RMSD calculation.

In addition, each protein residue’s fluctuation was calculated using root mean square fluctuation (RMSF) as per Eq 2.

$$RMSF_{i}=\sqrt{\frac{1}{T}}\overset{T}{\underset{t=1}{\sum }}{\left(r_{i}^{\prime }\left(t\right)-\;r_{i}\left(t_{ref}\right)\right)}^{2}$$ (2)

***T***: simulation interval; ***t***_***ref***_: reference time; ***r***_***i***_: position of the atoms under evaluation in frame x; $r_{i}^{'}$: position of the atoms in the reference frame.

### 2.5 Binding free energy calculation

The binding free energy for the complete trajectory (100 ns) was calculated using the MM/GBSA model of the Prime Schrödinger suite (Script-thermal_mmgbsa.py) [74–76]. This tool calculates the binding energy of protein and ligand by deploying molecular mechanics force field calculation and generalized born solvent model [77]. The molecular mechanics (MM) component represents the internal energy of the system consisting of bonded (bond energy, angle energy, dihedral energy) and non-bonded (van der Waal, electrostatic) interactions between protein and ligand. The change in bonded interaction in the free and bound state of protein and ligand is the same, implying ΔG_Bonded/Internal Energy_ ≈ 0. Thus, only non-bonded interaction contributed to ΔG. Moreover, the generalized born solvent model also consists of polar solvation energy (GB) and non-polar solvation energy (SA). Details of the binding free energy calculation are shown in Eq (3).


$$\Delta G_{Bind}=\;G_{Complex\left(minimized\right)}-\left(G_{\text{Receptor}\;\left(\text{minimized}\right)}-\;G_{\text{Ligand}\;\left(\text{minimized}\right)}\right)$$ (3)

$$\Delta {\text{G}}_{Bind}=\Delta \text{H}-\text{T}\Delta \text{S}\approx \Delta {\text{E}}_{gas}+\Delta {\text{G}}_{sol}-\text{T}\Delta \text{S}$$ (3.1)

$$\Delta E_{gas}=\Delta E_{\text{int}}+\Delta E_{ele}+\Delta E_{vdw}$$ (3.2)

$$\Delta G_{sol}=\Delta G_{GB}+\Delta G_{surf}$$ (3.3)

Δ*G*_Bind_: Change in Binding free energy.

*G*_*Complex*(*minimized*)_: Free energy of the complex

*G*_Receptor (minimized)_: Free energy for the receptor

*G*_Ligand (minimized)_: Free energy for the ligand

ΔH: Change in enthalpy

ΔS: Change in entropy (neglected in this equation ≈ 0)

Δ*E*_gas_: Change in gas phase interaction energy

Δ*E*_int_: Change in internal energy (no change as the same receptor and ligand is considered for the trajectory ≈ 0)

Δ*G*_sol_: Change in solvation energy

ΔG_GB_: Polar Solvation energy

Δ*G*_surf_: Non-polar solvation energy.

## 3. Results and discussions

### 3.1 Native ligand interactions

**Fig 2:**
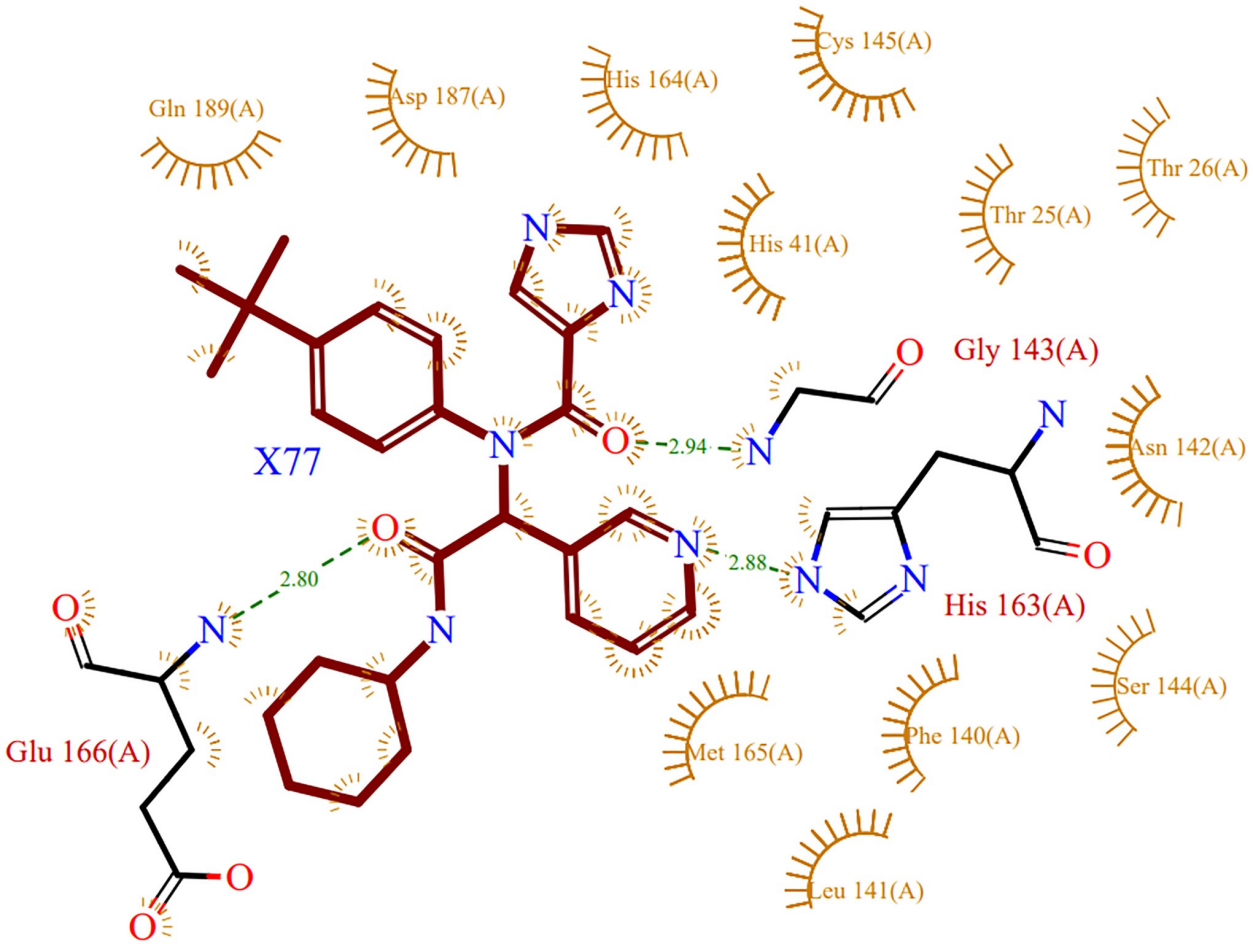
Native ligand (X77)-protein (PDB structure 6W63) 2D interactions plot: Representation of intermolecular interaction between native ligand and the active site amino acid residue of the target protein.

The native ligand (X77) co-crystalized with the M^pro^ was initially analysed for the native contacts as shown in Fig 2. It has been observed that catalytic dyads Cys145 and His41 were placed in the proximity of the ligand, which indicates the possible inhibitory properties of the ligand. Gly143, His163, and Glu166 formed hydrogen bonds with the ligand at 2.94Å, 2.88Å and 2.80Å hydrogen bond donor-acceptor distances.

### 3.2 Re-docking of native ligand

Native ligand X77 was removed from the PDB structure and redocked at the designated grid (description mentioned in the Methods section). The docking was performed using the Glide Schrödinger suite. This validated the docking protocol and used the native ligand as a control for further study. The best pose of the docked complex was studied to find the similarity between the native structure (PDB ID: 6W63) and the docked pose. In the native complex, it showed a total of 3 hydrogen bonds with Glu166, His163, and Gly143 each (Fig 2). However, in the best docked complex, Glu166 was retained while an additional Asn142 hydrogen bond was formed with the carboxyl oxygen of the ligand, which was initially bonded with Gly143 in its native form. His41, one of the catalytic dyad residues of SARS-CoV-2 M^pro^, is involved in a π-π stacking interaction with the benzene ring of the ligand. These observations set the docked pose of the native ligand to be used as a control.

### 3.3 Virtual screening

**Fig 3:**
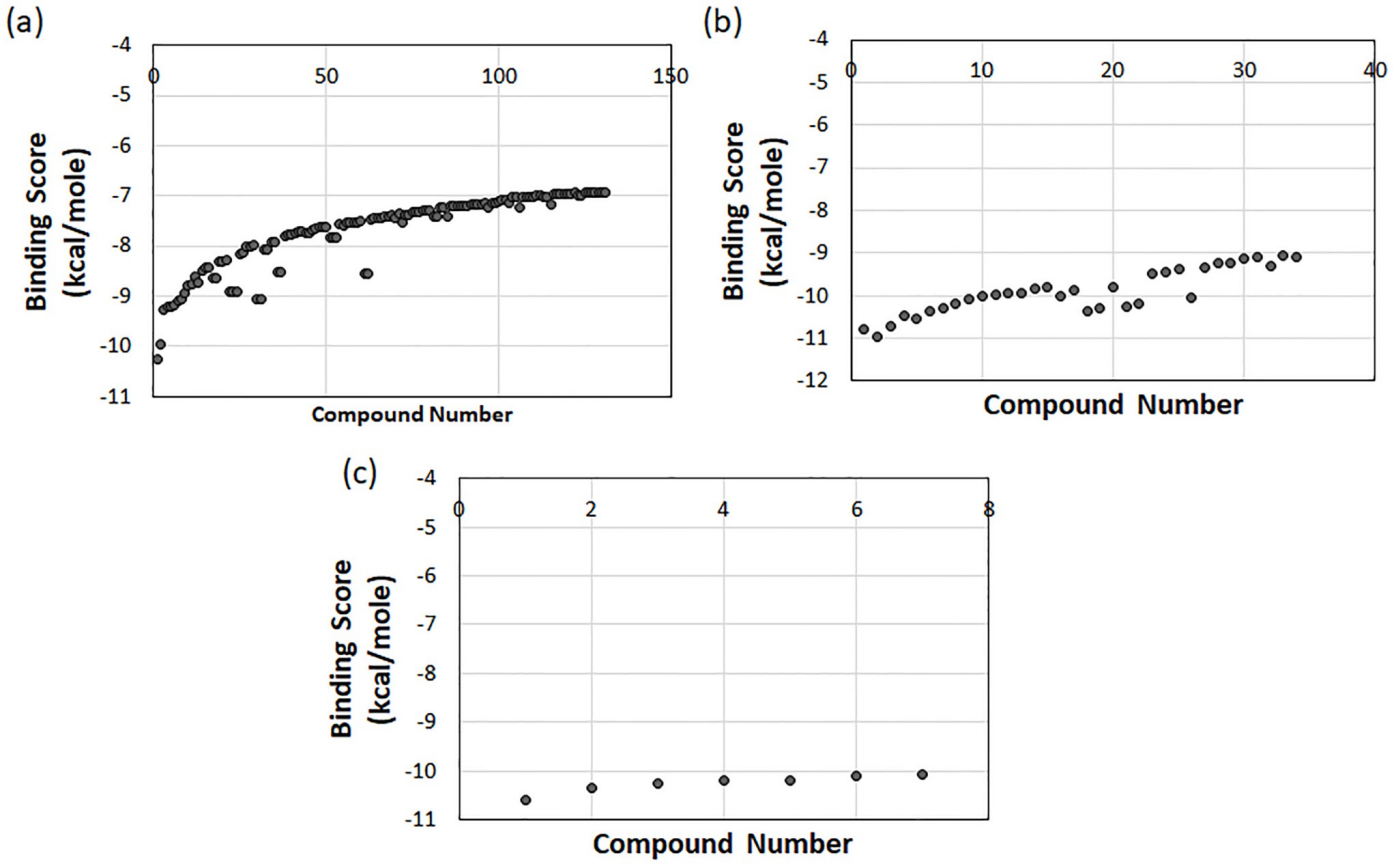
Binding scores of compounds were obtained via three different accuracy levels applied during the structure-based virtual screening process. (a) HTVS, (2) Glide SP, and (c) Glide XP.

This study used 330 anti-dengue compounds sourced from the DenvInD database. A list of these compounds is shown in S1 Table in S1 File. All these compounds have antiviral properties and thus could be potential compounds for screening against the SARS-CoV-2 M^pro^. These molecules were prepared in Ligprep to assign partial charge, radius, and van der Waal parameters. Each compound was used in multiple conformations to cover the possible relevant geometrical chemical space, and a total of 524 conformations were generated. These 524 conformers of 330 compounds that were initially screened using the HTVS (high-throughput virtual screening) protocol with 25% selection criteria. The result was 131 compounds for the next phase. In the next phase, Glide’s standard precision (SP) protocol was applied for the docking, and the top 25% of compounds were selected. It resulted in 34 compounds being selected for the next phase of docking using the extra precision (XP) protocol of Glide. Here, XP also used a 25% selection filter based on the binding score calculated in Glide XP. This resulted in 7 compounds with the maximum possibility of interacting with SARS-CoV-2 M^pro^ at its binding site. However, three of the seven compounds were the same with different conformations, so only the one with the highest binding score was used, while the other two were eliminated. This made the final bin with the top 5 unique compounds for further study. Fig 3 shows the binding score of selected compounds at each screening protocol. Fig 3(a) shows the 131 compounds selected after HTVS; none of the compounds had a binding score greater than -6.5 kcal/mol. The best binding score -10.25 kcal/mol was shown by (+-)-chartaceone D (Pubchem ID: 56834070), a flavonoid derivative.

Similarly, SP results is shown in Fig 3(b); here, 31 compounds were selected, and all showed binding energy better than -9.0 kcal/mol. In SP screening, the best binding score of -10.94 kcal/mol was shown by the tetrapeptide molecule H-Trp-Tyr-Cys-Trp-NH_2_ (Pubchem ID 118717692). Moreover, Fig 3(c) shows the last 7 compounds screened after XP screening, where all the compounds had a binding score better than -10.0 kcal/mol. Here again, (+-)-chartaceone D (Pubchem ID: 56834070) showed the best binding score of -10.61 kcal/mol. However, H-Trp-Tyr-Cys-Trp-NH_2_ (Pubchem ID 118717692) showed the best binding score in Glide SP was eliminated after Glide XP screening.

**Fig 4:**
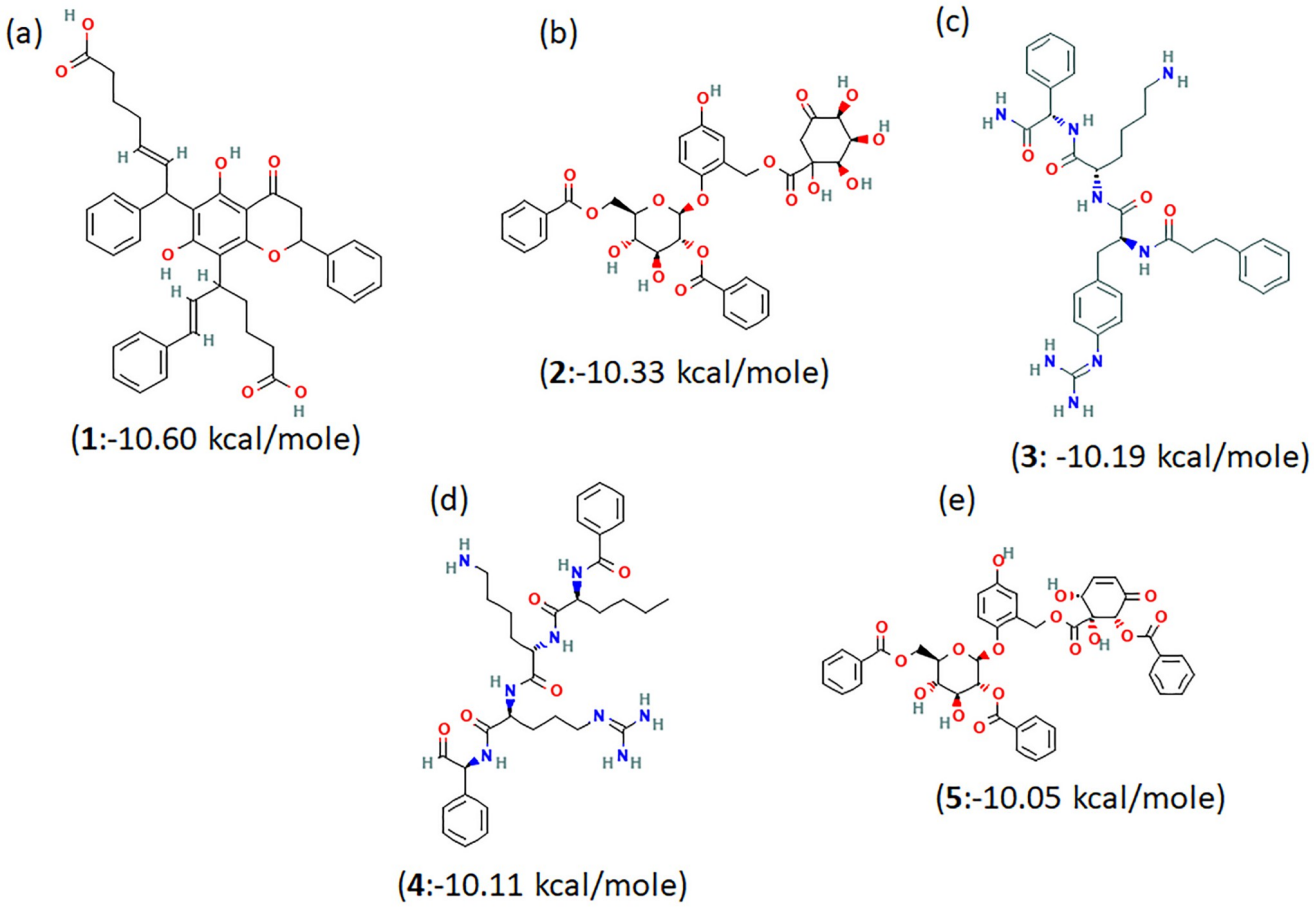
2D chemical structures of top 5 compounds obtained via HTVS, SP and XP screening with their binding score. (a) CHEMBL1940602 (b) CHEMBL2036486 (c) CHEMBL3628485 (d) CHEMBL200972 and (e) CHEMBL2036488.

As discussed, five unique compounds were selected after performing all three screening protocols, as shown in Fig 4 with their corresponding binding scores. These compounds are:

1. Pubchem ID 56834070: (E)-7-[8-[(E)-6-carboxy-1-phenylhex-1-en-3-yl]-5,7-dihydroxy-4-oxo-2-phenyl-2,3-dihydrochromen-6-yl]-7-phenylheptv-5-enoic acid (+/-Chartaceone D)—labelled as CHEMBL1940602
2. Pubchem ID 57409246: [2-[(2S,3R,4S,5S,6R)-3-benzoyloxy-6-(benzoyloxymethyl)-4,5-dihydroxyoxan-2-yl]oxy-5-hydroxyphenyl]methyl (2R,3S,4S)-1,2,3,4-tetrahydroxy-5-oxocyclohexane-1-carboxylate (Flacourtoside D)—labelled as CHEMBL2036486
3. Pubchem ID 122193488: (2S)-6-amino-N-[(1S)-2-amino-2-oxo-1-phenylethyl]-2-[[(2S)-3-[4-(diaminomethylideneamino)phenyl]-2-(3 phenylpropanoylamino) propanoyl] amino] hexanamide (deamino-Phe-Phe(4-guanidino)-Lys-Phg-NH2)—labelled as CHEMBL3628485
4. Pubchem ID 11974638: N-[(2S)-1-[[(2S)-6-amino-1-[[(2S)-5-(diaminomethylideneamino)-1-oxo-1-[[(1S)-2-oxo-1-phenylethyl]amino]pentan-2-yl] amino]-1-oxohexan-2-yl]amino]-1-oxohexan-2-yl]benzamide (Bz-Nle-Lys-Arg-Phg-al)—labelled as CHEMBL200972
5. Pubchem ID 57409350: [2-[(2S,3R,4S,5S,6R)-3-benzoyloxy-6-(benzoyloxymethyl)-4,5-dihydroxyoxan-2-yl]oxy-5-hydroxyphenyl]methyl (1R,2R,6R)-6-benzoyloxy-1,2-dihydroxy-5-oxocyclohex-3-ene-1-carboxylate (Flacourtoside F)—labelled as CHEMBL2036488

### 3.4 ADME and toxicity analysis

The proposed molecules as therapeutics must be biologically active with minimum toxicity. Therefore, the selected compounds were studied for their pharmacokinetic features such as absorption, distribution, metabolism, excretion (ADME) and toxicity. Most drugs fail due to their least biological availability and higher toxicity at the clinical trial stages; therefore, assessing those parameters at the early stage of drug discovery is mandatory. The selected antiviral compounds were the non-inhibitors of cytochromes (CYP1A2, CYP2C19, CYP2C9, CYP2D6, and CYP3A4) which play an essential role in the metabolism of drugs and xenobiotics. All selected compounds were found to lack blood-brain barrier (BBB) features and lower gastrointestinal (GI) absorption, marked as an essential feature of drug molecules. The selected compounds were shown violations to the rules such as Lipinski, Ghose, Veber, Egan, and Mugan, which are considered an important rule of thumb to evaluate drug likeliness. However, these rules are not required to be followed strictly as several drugs in the past have violated these rules but still got FDA approval [78]. Among all five selected compounds, only CHEMBL1940602 showed an acceptable bioavailability score (0.56). Notably, All five compounds were found to be non-mutagenic as well as non-carcinogenic. Other pharmacological and drug-likeliness features are also enlisted in **S2** and **S3** Tables S1 File. Overall, the selected compounds obeyed the important ADMET parameters and can be considered for further studies.

### 3.5 Docked pose interaction

**Fig 5:**
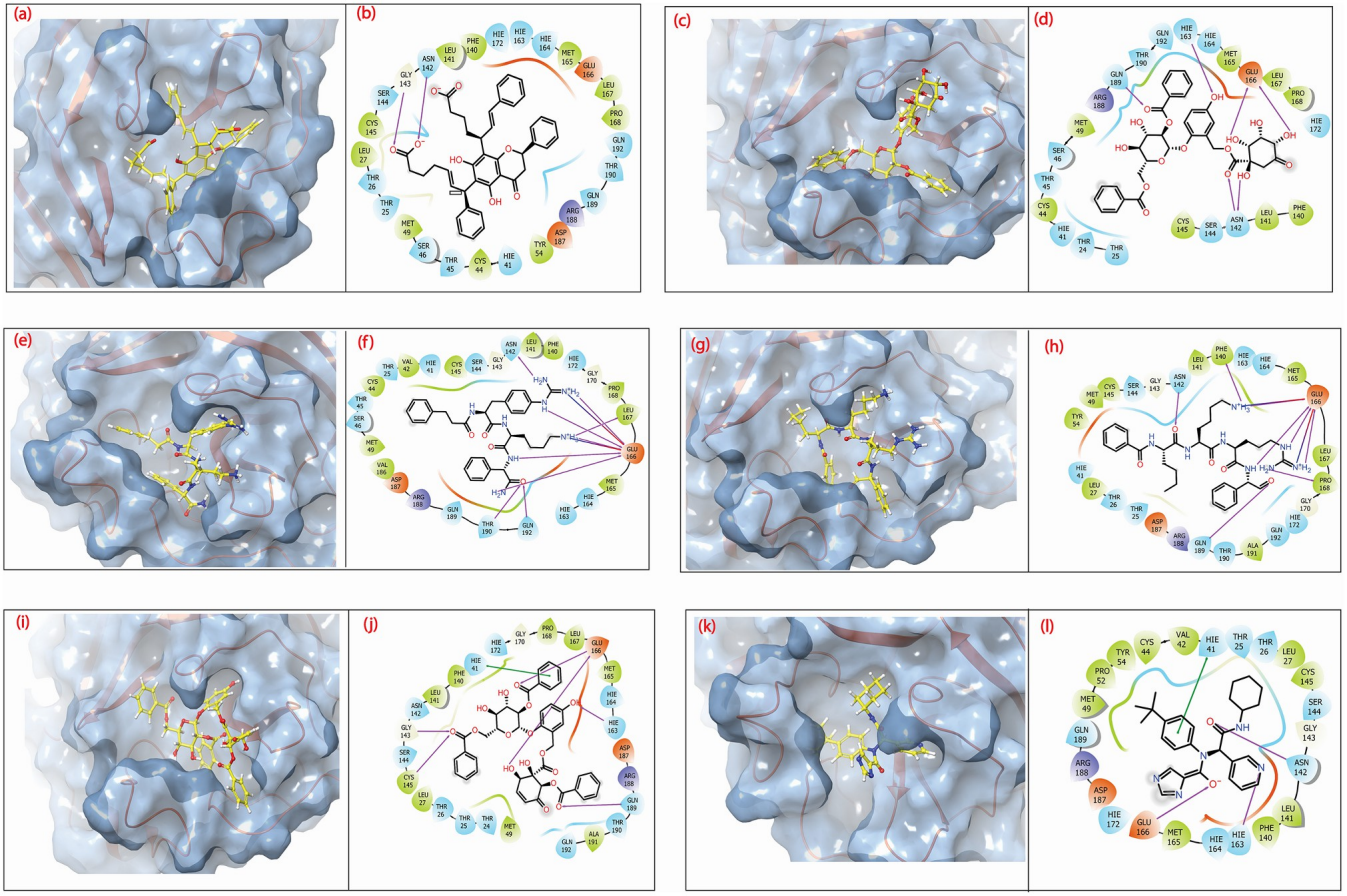
3D and 2D interaction profiles for SARS-CoV-2 M^pro^- compounds (a-b) M^pro^-CHEMBL1940602 (c-d) M^pro^-CHEMBL2036486 (e-f) M^pro^-CHEMBL3628485 (g-h) M^pro^-CHEMBL200972 (i-j) M^pro^-CHEMBL2036488, and (k-l) M^pro^-X77 (control).

Screened molecules were further analysed for their interactions with SARS-CoV-2 M^pro^ protein through its binding site residues, as shown in Table 1 and Fig 5. Compound **1** (Fig 5(a) and 5(b)) has formed hydrogen bonds with Gly143 and Asn142. Both these residues form hydrogen bonds in the control compound (X77), Gly143 in the crystal structure and Asn142 in the best-docked pose of X77. CHEMBL2036486 (Fig 5(c) and 5(d)) has the possibility of forming four hydrogen bonds with Gln189, His163, Glu166, and Asn142. It also contains two similar H-bond interactions shown with the control compound (X77); these are Glu166 (native form) and Asn142 (docked pose).

**Table 1:** Intermolecular interactions noted for the screened compounds with the SARS-CoV-2 M^pro^ within 4 Å around the docked ligand in the respective binding pockets.

Compounds	H-bond	Hydrophobic	Polar	π-π/ *π-cation	Salt bridge	Positive	Negative
CHEMBL1940602	Asn^142^, ,Gly^143^,	Ley^27^, Cys^44^, Met^49^, Phe^140^, Leu^141^,Cys^145^, Met^165^, Leu^167^,Pro^168^.	Thr^25^,Thr^26^, His^41^, Thr^45^, Ser^46^,Asn^142^, Ser^144^,His^163^, His^164^, His^172^, Gln^189^, Thr^190^, Gln^192^,	--	--	Arg^188^	Glu^166^, Asp^187^
CHEMBL2036486	Asn^142^, His^163^, Glu^166^, Gln^189^	Cys^44^, Met^49^, Phe^140^, Leu^141^,Cys^145^, Met^165^, Leu^167^,Pro^168^	Thr^24^, Thr^25^, His^41^, Thr^45^, Ser^46^,Asn^142^, Ser^144^,His^163^, His^164^, His^172^, Gln^189^, Thr^190^	--	--	Arg^188^	Glu^166^
CHEMBL3628485	Asn^142^, Glu^166^, Leu^167^, Thr^190^, Gln^192^	Val^42^, Cys^44^, Met^49^, Phe^140^, Leu^141^,Cys^145^, Met^165^, Leu^167^,Pro^168^, Val^186^	Thr^25^, His^41^, Thr^45^, Ser^46^, Asn^142^, Ser^144^, His^163^, His^164^, His^172^, Gln^189^, Thr^190^, Gln^192^	--	Glu^166^	Arg^188^	Glu^166^, Asp^187^
CHEMBL200972	Phe^140^, Asn^142^, Glu^166^, Pro^168^, Gln^189^	Ley^27^,Met^49^, Tyr^54^,Phe^140^, Leu^141^,Cys^145^, Met^165^, Leu^167^,Pro^168^	Thr^25^,Thr^26^, His^41^, Asn^142^, Ser^144^,His^163^, His^164^, His^172^, Gln^189^, Thr^190^, Gln^192^,	--	Glu^166^	Arg^188^	Glu^166^, Asp^187^
CHEMBL2036488	Gly^143^, Cys^145^, His^193^, Glu^166^, Gln^189^	Ley^27^,Met^49^,Phe^140^, Leu^141^,Cys^145^, Met^165^, Leu^167^,Pro^168^,Ala^191^	Thr^24^, Thr^25^,Thr^26^, His^41^,Asn^142^, Ser^144^,His^163^, His^164^, His^172^, Gln^189^, Thr^190^, Gln^192^,	His^41^	--	Arg^188^	Glu^166^, Asp^187^
X77 (Control)	Asn^142^, His^163^, Glu^166^	Ley^27^, Val^42^, Cys^44^, Met^49^, Pro^52^,Thr^54^, Phe^140^, Leu^141^,Cys^145^, Met^165^,	Thr^25^,Thr^26^, His^41^, Asn^142^, Ser^144^,His^166^His^164^, His^172^, Gln^189^	His^41^	--	Arg^188^	Glu^166^, Asp^187^

In CHEMBL3628485 (Fig 5(e) and 5(f)), Glu166 showed the potential to form 6 H-bonds on its own, while Asn142, Thr190, and Gln192 each have the potential to form one H-bond. In terms of polar interaction, it was most likely that CHEMBL3628485 (Fig 5(g) and 5(h)) would interact strongly with M^pro^ CHEMBL200972 also showed similar characteristics as CHEMBL3628485 and Glu166 showed the possibility to interact at five sites of the ligand with H-bonds. However, Phe140, Asn142, Pro168, and Gln189 were additional residues that formed 1 H-bond each with CHEMBL200972. Similar to CHEMBL1940602, CHEMBL2036486, and CHEMBL3628485, two residues (Glu166 and Asn142) had overlapping H-bond formation with control in their crystal and docked poses, respectively. CHEMBL2036488 (Fig 5(i) and 5(j)) had a total of 6 H-bonds and 1 Π-Π stacking interaction. The stacking interaction formed by His41 (catalytic dyad residue) with the benzene ring of CHEMBL2036488 was also found in the best pose of the control compound (Fig 5(k) and 5(l)). The control compound has H-bond interactions with Glu166 and Gly143 (Figs 2, 5(k) and 5(l)), which were also found in CHEMBL2036488. Another catalytic dyad residue (Cys145) also formed an H-bond with CHEMBL2036488. Additionally, Gln189 formed an H-bond similar to CHEMBL3628485.

### 3.6 Molecular dynamic (MD) simulation

**Fig 6:**
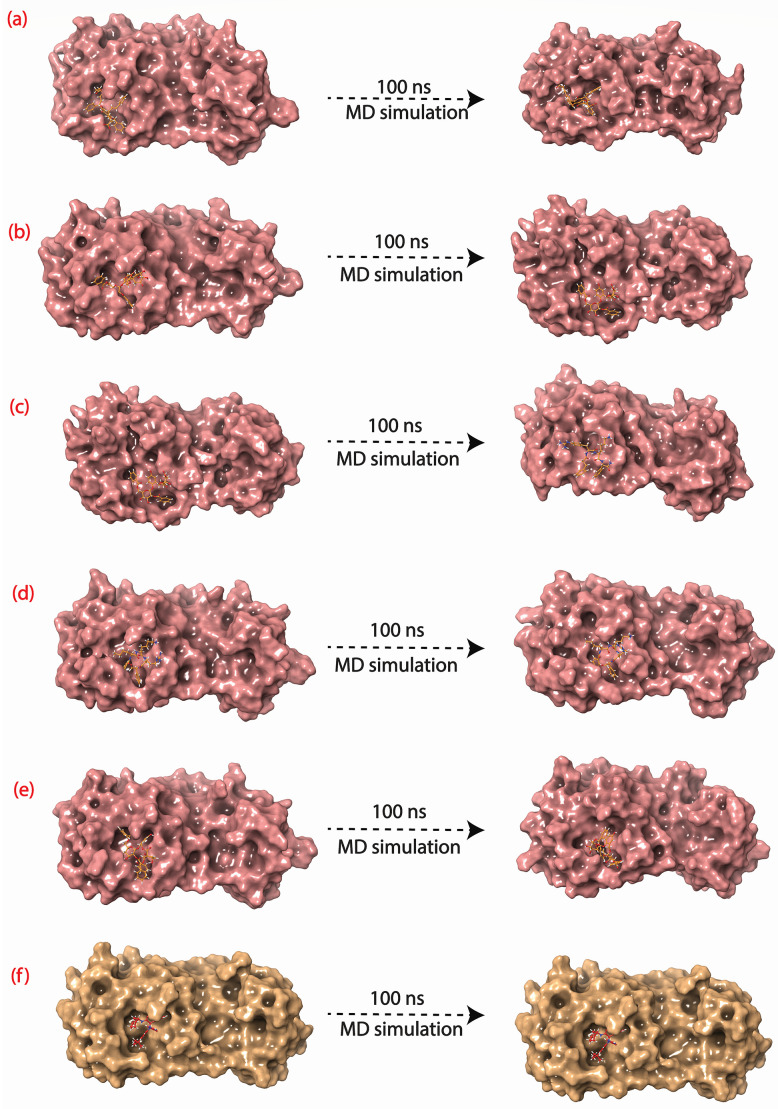
Dynamic flexibility depiction using first and last pose obtained from MD simulation for (a) M^pro^-CHEMBL1940602 (b) M^pro^-CHEMBL2036486 (c) M^pro^-CHEMBL3628485 (d) M^pro^-CHEMBL200972 (e) M^pro^-CHEMBL2036488, and (f) M^pro^-X77 (control).

Molecular dynamic simulation is an essential computational technique to establish the dynamic stability of protein-ligand interactions of docked complexes with respect to time. Best docked poses of all screened and control compounds were used for 100 ns explicit MD simulation at 300 K temperature and 1 bar pressure. Later, the MD was also performed at 310 K (Human body temperature), and the results are given in S2 File. MD trajectory of 100 ns was generated for all the selected complexes. Dynamic stability is shown in Fig 6 and **S3 Fig** in S1 File, where the initial pose (first frame of simulation) and final pose (last frame of simulation) are depicted. CHEMBL1940602, CHEMBL2036486, and CHEMBL200972 showed higher conformational consistency at the binding site, whereas CHEMBL3628485 and CHEMBL2036488 showed considerable translation and rotational movement to attain a more thermodynamically stable state. The control compound (Fig 6(f)) demonstrated the higher stability with the least amount of translation and rotation movement.

#### 3.5.1 Root Mean Square Deviation (RMSD)

**Fig 7:**
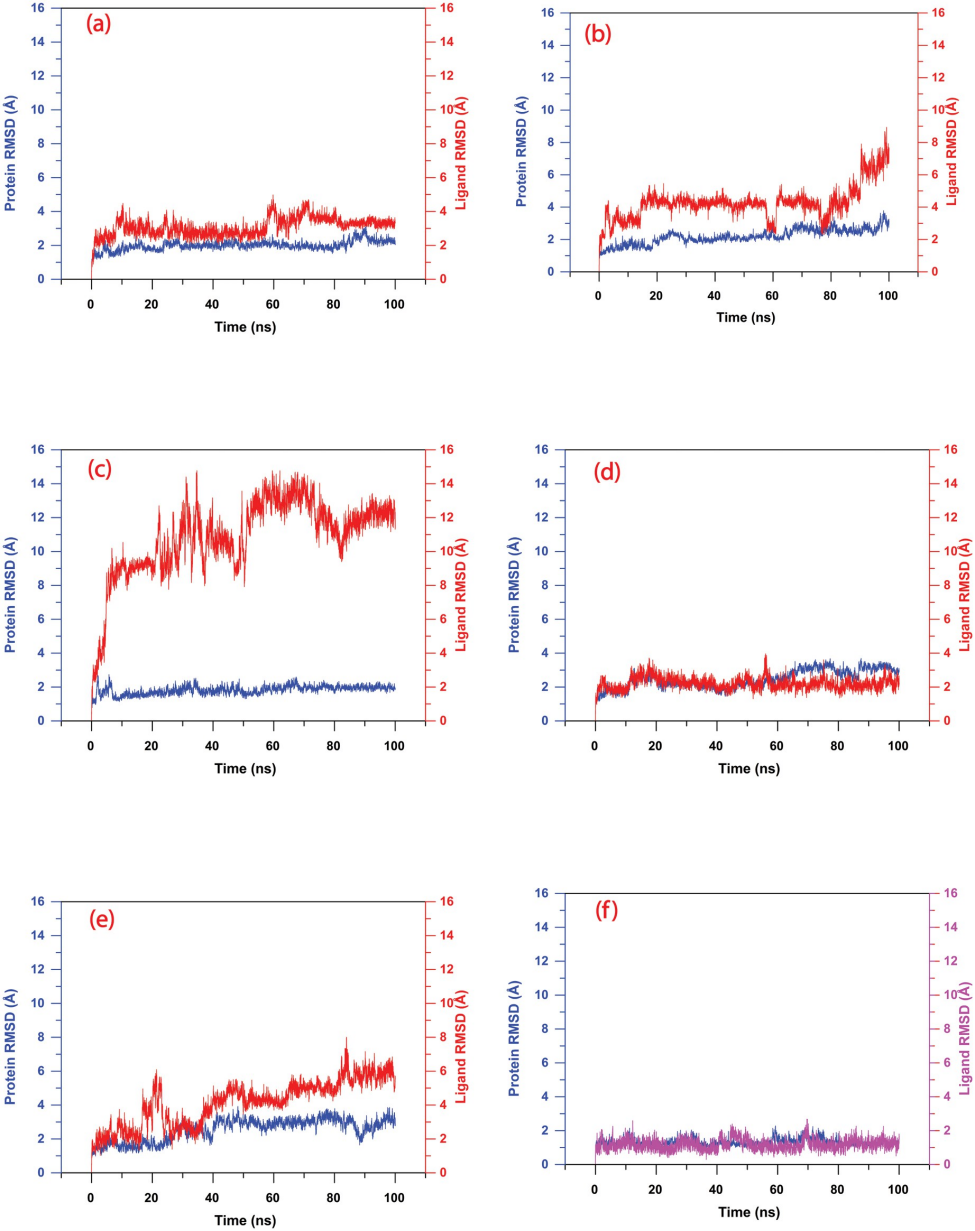
Root mean square deviation (RMSD) plots for (a) M^pro^-CHEMBL1940602 (b) M^pro^-CHEMBL2036486 (c) M^pro^-CHEMBL3628485 (d) M^pro^-CHEMBL200972 (e) M^pro^-CHEMBL2036488, and (fCH) M^pro^-X77 (control), calculated over the period of 100 ns MD simulation.

RMSD over the trajectory measures the deviation of the system with respect to the first frame (original) state. Here, RMSD was computed over the whole trajectory for both protein and ligand separately to measure the equilibration stage of the protein-ligand complex. The conformational variance of less than 3 Å is deemed acceptable. If there is a minimum deviation, then the lines in the RMSD plot are parallel to the X-axis. Fig 7 shows the RMSD of all screened and control compounds over the 100 ns trajectory. Protein RMSD in all cases was under 3 Å, and thus it did not show any significant deviation. Control shows the minimum deviation for protein and ligand <2 Å. Here, both Ligand^RMSD^ and Protein^RMSD^ were stable from the beginning of the simulation and maintained the same pattern until the completion of the experiment. Observing that both lines (protein and ligand) are parallel to the X-axis demonstrates their higher conformational stability. CHEMBL200972 showed the minimum deviation (<3 Å) pattern among the screened compounds and attained stability at the very early stage of the simulation. Also, CHEMBL1940602 took 10 ns to reach stability, exhibited a steady pattern and showed an overall deviation of less than 4 Å. It deviated at 10 ns and between 60 to 80 ns up to 4 Å, and then it came down to attain the equilibrium at 3 Å. Among all the screened compounds, CHEMBL3628485 showed the highest deviation up to 14 Å which cannot be considered as an acceptable RMSD. The translation motion of CHEMBL3628485was also observed in the first and last pose obtained from simulation (Fig 6), confirming its higher dynamic instability. These compounds deviated up to 8 Å as soon as the simulation started and never attained a stable trajectory ending with a maximum deviation at ≥14 Å accompanied by continuous fluctuation till the end of the simulation. This clearly suggests that the compound is not stable at the binding site, and a more extended simulation (> 100 ns) would be required to understand the behaviour of CHEMBL3628485. Compound CHEMBL2036486 had a stable trajectory for the initial 75 ns of simulation; however, it showed deviation up to > 8 Å. Like CHEMBL3628485, CHEMBL2036486 would also be required longer (>100 ns) simulation studies to understand whether it is stable in the binding pocket of protein or not. However, in the case of CHEMBL2036488, the deviation is not high as CHEMBL3628485 but also not stable as CHEMBL1940602, CHEMBL200972, and control compound complexes. Here, it deviated to RMSD greater than 4 Å after 40 ns of simulation time and then further deviated to 6 Å during the last 20 ns of simulation. In conclusion, compared to the control, the dynamic stability shown by the screened was in the decreasing order of CHEMBL200972, CHEMBL1940602, CHEMBL2036486, CHEMBL2036488, and CHEMBL3628485.

#### 3.5.2 Root Mean Square Fluctuation (RMSF)

As noted previously, protein exhibited a steady pattern; therefore, none of the complexes exhibited abrupt RMSF behaviour, as illustrated in **S1 Fig in** S1 File. Protein RMSF values were calculated for the Cα atoms of each residue. All residues from M^pro^ showed stable RMSF < 3 Å in all the complexes. Only in the CHEMBL200972 docked complex, there was a single relatively higher peak shown for Ala194 where RMSF > 3 Å. The control compound among all the docked complexes showed the most stable pattern for all the protein residues. None of the key residues displayed a substantial conformational change in this instance. This reflects the stability of the protein in the protein-ligand complex.

### 3.6 Binding free energy calculation

To assess the stability of the complex in MD simulation, the binding free energy of MM/GBSA was computed throughout the 100 ns trajectory. Table 2 shows the MM/GBSA binding free energy of all five compounds and the control compound. ΔG_bind_ is minimum for CHEMBL200972that shows it maximum binding affinity, -88.98 kcal/mol with M^pro^. However, CHEMBL2036486 (-70.13 kcal/mole) and CHEMBL2036488 (-72.84 kcal/mol) showed better performance than control and were considered relatively acceptable complexes as compared to the control compound-complex. Compound CHEMBL1940602and control exhibited similar ΔG_bind_ (-64.72 and -64.53 kcal/mol); thus, they cannot be differentiated. Furthermore, MM/GBSA data showed that CHEMBL3628485had relatively poor binding (-40.89 kcal/mol) with M^pro^; thus, the compound is not highly stable at the protein’s binding site. Van der Waal interaction was the major contributor to binding free energy for CHEMBL1940602 (-76.30 kcal/mol), CHEMBL2036486 (-56.04 kcal/mol), CHEMBL2036488 (-61.69 kcal/mol) and control (-50.86 kcal/mol). In contrast, CHEMBL3628485 and CHEMBL200972were maximally stabilised by coulombic interaction with -85.21 kcal/mol and -115.93 kcal/mol energies, respectively. The best H-bond energy was found best in compound CHEMBL200972 at -4.38 kcal/mol, followed by CHEMBL3628485, CHEMBL2036486, CHEMBL2036488, control, and CHEMBL1940602 at -2.94 kcal/mol, -2.30 kcal/mol, -2.04 kcal/mol, -1.88 kcal/mol, and -1.55 kcal/mol, respectively.

**Table 2:** MM/GBSA binding energy components for selected and control compounds.

MM/GBSA components	CHEMBL1940602	CHEMBL2036486	CHEMBL3628485	CHEMBL200972	CHEMBL2036488	Control (X77 inihbitor)
**ΔG_Bind_**	-64.72±9.23	-70.13±10.00	-40.89±11.28	-88.98 ± 8.95	-72.84±11.53	-64.53±7.26
**ΔG_Bind Coulomb_**	-2.82±38.59	-28.23 ± 8.04	-85.21±25.94	-115.93 ±23.70	-26.07 ± 7.48	-33.01 ± 25.05
**ΔG_Bind Covalent_**	7.10±2.27	2.30 ± 2.95	3.46± 2.62	8.12 ± 2.83	3.09 ± 4.20	3.52 ± 1.83
**ΔG_Bind Hbond_**	-1.55±1.10	-2.30 ± 0.76	-2.94± 1.17	-4.38 ± 0.58	-2.04 ± 0.66	-1.88 ± 0.37
**ΔG_Bind Lipo_**	-19.86±1.64	-16.10 ± 2.28	-9.28± 2.89	-19.47 ± 1.77	-17.28 ± 2.95	-14.58 ± 1.11
**ΔG_Bind Packing_**	-2.59±0.58	-2.23 ± 0.78	-0.73 ± 0.87	-1.93 ± 0.69	-2.39 ± 0.89	-1.24 ± 0.44
**ΔG_Bind Solv GB_**	17.76±34.60	32.56 ± 5.15	85.51 ± 22.06	109.30 ± 21.63	33.54± 5.12	33.52± 19.90
**ΔG_Bind vdW_**	-76.30±3.72	-56.04 ± 5.69	-31.83 ± 7.58	-65.33± 4.32	-61.69 ± 7.77	-50.86 ± 2.85
**StrainEnergy**	7.14± 2.499	9.10 ± 3.75	6.55 ± 4.25	14.17± 2.93	12.28 ± 4.50	5.99 ± 2.61

### 3.7 Interactive residues in MD trajectory

Residues that showed interaction with protein in their best-docked poses were also analysed for their interaction over the period of 100 ns of simulation. Compound CHEMBL1940602 had a stacking interaction with catalytic dyad residue His41, which was retained for 58% of the simulation time. However, water-mediated H-bonds by Asp187 and His164 were shown for 91% and 64%, respectively. Here, H-bond interaction with Asp^187^ is a strong indicator for the stabilization of CHEMBL1940602 in the binding site of M^pro^. In compound CHEMBL2036486, Glu166 formed a water bridge with the ligand’s hydroxyl group and had a maximum occurrence frequency of 74% during the simulation. CHEMBL3628485 formed H-bonds with Glu47 and Glu166, but the frequency of occurrence was < 60% in both cases, indicating that CHEMBL3628485 has lower interaction stability with the pocket lining residue of M^pro^. In contrast, CHEMBL200972 had several H-bond interactions where the frequency of occurrence was > 60%, especially with the residue Glu166 (84%, 72%, 72%, 87% and 81%), His164 (69%) and Gln189 (76%). Glu166 was found as a crucial residue in the experimental co-complex of the native ligand with the protein. This indicates the higher stability of CHEMBL200972 within the binding site of M^pro^.

**Fig 8:**
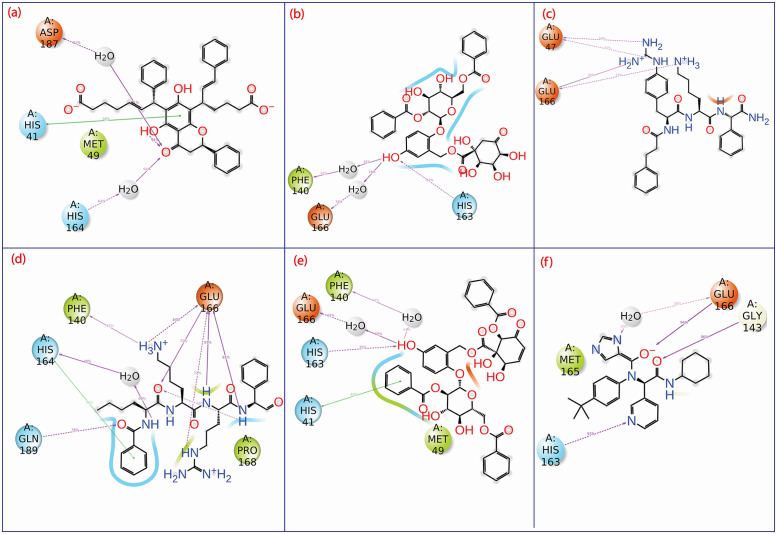
2D interaction diagram of protein-ligand interactions maps for (a) M^pro^-CHEMBL1940602 (b) M^pro^-CHEMBL2036486 (c) M^pro^-CHEMBL3628485 (d) M^pro^-CHEMBL200972 (e) M^pro^-CHEMBL2036488, and (f) M^pro^-X77 (control), over the 100 ns MD simulation.

Similarly, the CHEMBL2036488 complex also had Glu166, and His163 which showed H-bonds with ligand for more than 60% of the entire frame (60% and 70%). This also indicated a higher and relevant binding stability of CHEMBL2036488 at the protein’s binding site. Protein-ligand interaction and percentage of occurrence over the 100 ns simulation trajectory is shown in Fig 8 and **S2 Fig in** S1 File. As it can be observed in Fig 8(f), the control ligand started showing H-bond with Gly143 for 86% of the total time frame, which was missing in the rigid docked complex. The control compound also showed a considerable binding with Glu166 with a 94% frequency of occurrence of H-bonds. His163, a critical contact in the native ligand in the crystal structure (Fig 2), was also missing from the docked complex of control (Fig 5(f)). However, it gained access to the ligand during simulation, and Fig 8(f) shows the His163 with 93% of the time involved in H-bond.

### 3.8 Principal component analysis

**Fig 9:**
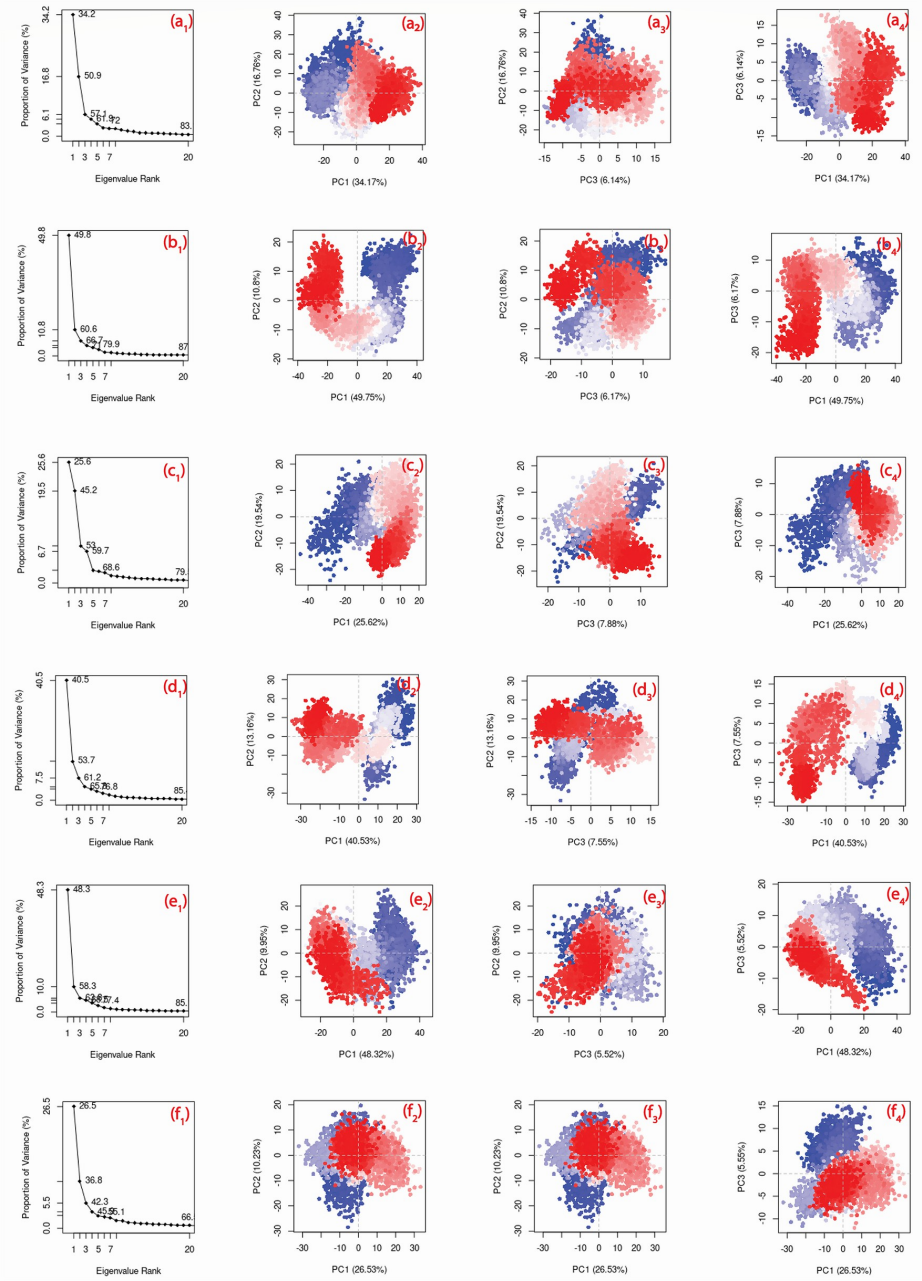
Principal component cluster analysis along first three PCs for (a1-a4) CHEMBL1940602, (b1-b4) CHEMBL2036486, (c1-c4) CHEMBL3628485, (d1-d4) CHEMBL200972, (e1-e4) CHEMBL2036488 and (f1-f4) X77 (control). The spread of dots represents the degree of conformational change while the colour code represents the time steps (blue: initial, white: intermediate and red: final).

PCA is utilised to handle the high dimensional movement of the system by minimising its space during the MD simulation trajectory. This was achieved by transforming every set of associated atomic motions into a collection and assessment of principal components. These principal components are the true representatives of the motion of the biological system during the MD simulation. The PCA was used to evaluate the association between statistically significant conformational variations created during the MD simulation. This study considered the first three principal components to show the conformational variation. Compound CHEMBL1940602 showed a 61.9% cumulative variation for the top 3 PCAs. Similarly, for compounds CHEMBL2036486, CHEMBL3628485, CHEMBL200972, CHEMBL2036488, and control, the top 3 PCAs constituted 66.7%, 59.7%, 61.2%, 63.8% and 45.56%, respectively. Fig 9 shows the top 3 PCA for all 5 compounds and control. Here, each dot represents the conformation of a protein. The spread of different colours in the plot depicts the degree of conformational change. However, the change in colour (blue to white to red) represents the simulation’s initial, intermediate, and final time step. Fig 9(a2) shows the first two principal component analyses for CHEMBL1940602 with a wide range of data points that reflect the degree of conformational changes recorded during the simulation.

Moreover, the data points were found continuous in CHEMBL1940602 for all the PCs plots that showed the constant conformational change. The conformation of compound CHEMBL2036486 varied similarly, but there was a non-overlapping subspace in the PC1-PC2 plot that indicated the distinct periodic jump. Compounds CHEMBL3628485, CHEMBL2036488, and control showed similar patterns of uniform conformational change. However, the uniformity was maximum in the control case. Compound CHEMBL200972 showed a relatively lesser distribution in the PC1-PC2 plot, which indicates a lower degree of conformational change.

In each docked complex, M^pro^ structures show a significant drop in variance about the first three eigenmodes, suggesting that docked ligands have induced conformational motion. The variance was followed by the elbow point, but no further significant changes in eigen fraction were observed after 4 to 20 eigen values (Fig 9). This observation suggested that the initial stages of simulation showed considerable flexibility in protein structure docked with respective ligands, but this flexibility reduced with simulation intervals. It is further suggested that the local fluctuations in M^pro^ structure in each docked complex tend to stabilize with time, as is suggested by the decreasing percentage of eigen modes. Previous studies have observed similar results with fructose transporter GLUT5 and G-protein-coupled receptor 119 docked with their respective ligands [79, 80].

Three PCs or eigen vectors for M^pro^ docked with selected putative compounds and X77 (control) were extracted from the respective MD trajectories and represented in clusters (Fig 9). The analysis of all three PCs supported the compact-cluster motions in Mpro in respective complexes during the MD simulation. Overall, the coupled motion in Mpro structure across all the system demonstrated the stiffness and significant fluctuations induced at the active site on binding of selected ligands during the simulation. Hence the overall observations suggested the stability of selected compounds i.e., CHEMBL1940602, CHEMBL2036486, CHEMBL3628485, CHEMBL200972, and CHEMBL2036488 against X77 inhibitor in the Mpro active pocket, and finally restricting the essential motion of protein required for enzymatic function, leading to its inhibition.

## 4. Discussions

This work demonstrates the structure-based screening of anti-dengue drugs against the SARS-CoV-2 M^pro^. In SARS-CoV-2, the main protease (M^pro^) is responsible for the maturation of polyprotein, which is essential for the virus’s proliferation [20]. In addition, M^pro^ also plays a role in the proteolytic processing of enzymes required for the replication of SARS-CoV-2 [81, 82]. This makes M^pro^ an essential pharmacological target for suppressing SARS-CoV-2 proliferation, which contains the catalytic dyad (Cys145 and His41) in a substrate binding cleft required for its catalytic function. The virtual screening of experimentally validated 330 anti-dengue compounds known for their antiviral activity resulted in the identification of potential Mpro active site binders named CHEMBL1940602, CHEMBL2036486, CHEMBL3628485, CHEMBL200972, and CHEMBL2036488. The computational approaches such as high throughput virtual screening, molecular docking, and molecular dynamic simulation, were implemented in this study for the in-silico characterization of known anti-DENV compounds against SARS-Cov-2 M^pro^. The results obtained from all these computational experiments were compared to the control M^pro^ ligand, i.e. X77, derived from the crystal structure of SARS-CoV-2 Mpro (PDB ID:6W63). The amino acids Gly143, Glu166, Cys145, and His163 are reported as critical binding site residues involved in forming H-bonds with the ligand [41], which was also observed in the case of the control ligand (X77), found to form H-bonds with Gly143 and Glu166. The substrate binding site consists of four subsites S1’, S1, S2, and S4, [24–28]and the binding of chemical compounds to these subsites leads to the inhibition of M^pro^.

A catalytic dyad (Cys145 and His41) creates a nucleophilic reaction required for the proteolytic cleavage [83] in which a proton is translocated from Cys145 to His41 due to simultaneous nucleophilic attack on the peptide carbonyl carbon by the sulfur atom of Cys145. Further, the proton from His145 is translocated to the substrate nitrogen atom resulting in the formation of acyl-enzyme complex intermediate, which assists peptide cleavage [84, 85].

The Initial docking poses for all screened compounds showed that Glu166 had been observed as a potential hydrogen bond formation residue in four of five selected compounds. Glu166 has also been reported to interact with protease inhibitors such as telaprevir, boceprevir, and narlaprevir in the case of HCV NS3/4A proteolytic cleavage [86]. The alignment of earlier findings with the outcome of this study indicates the possible inhibitory activities of screened compounds (CHEMBL2036486, CHEMBL3628485, CHEMBL200972, and CHEMBL2036488). However, a protein-ligand complex containing CHEMBL1940602 showed the involvement of Asn142 in forming H-bonds. An earlier study found that Asn142 was in the list of residues that formed H-bonds with 17 Marine Natural Products, which were found to be M^pro^ inhibitors [87].

Additionally, Gly143 was reported as the most potent residue to form H-bonds [41], and this study showed its presence in the complex containing CHEMBL1940602 as an H-bond acceptor. Compound CHEMBL2036488 formed π-π interaction with catalytic residue His41. This interaction was also reported in the case of baicalein, which exhibited a distinctive interaction pattern with SARS-CoV-2-M^pro^ [88].

The best-docked poses of all five screened compounds were further analysed for their dynamic stability within the docked complex via MD simulation. In all the docked complexes, the α-Carbon atoms of SARS-CoV-2 Mpro showed fluctuations in the considered range (< 3 Å) compared to the control docked complex. Furthermore, All protein fit ligands except CHEMBL3628485 showed a stable trajectory with an RMSD value (< 3 Å) which is considered highly stable as reported in several previous studies [89–91]. Protein-ligand interaction study over the simulation period confirmed that most of the interactions shown in the docked poses were retained during the MD simulation. Glu166 was the most common residue that showed interaction in the case of all the screened compounds except CHEMBL1940602 over the entire simulation trajectory. The compound CHEMBL200972 showed the most stable trajectory and formed an H-bond with Glu166 as the maximum number of simulation frames. Along with the high dynamic stability, the binding free energy of CHEMBL200972 was -88.98 ± 8.95 kcal/mole, which proved it the most energetically stable compound among all protein-ligand complexes over the 100 ns simulation. However, the remaining four molecules also showed relevant ΔG values compared to the control molecule (X77).

## 5. Conclusion

The Repositioning of anti-dengue compounds against SARS-CoV-2 M^pro^ could facilitate the discovery of potential anti-SARS-CoV-2 therapeutics. This study showed a rapid method to screen a library of 330 anti-dengue compounds against the substrate binding site of SARS-CoV-2 M^pro^. It evaluated the findings by comparing them with Crystal ligand structure (PDB ID: 6W63) X77. Based on the docking score (< 10.0 kcal/mol), the top 5 compounds; a) Chartaceone D (CHEMBL1940602) (b) Flacourtoside D (CHEMBL2036486), (c) deamino-Phe-Phe(4-guanidino)-Lys-Phg-NH_2_ (CHEMBL3628485) (d) Bz-Nle-Lys-Arg-Phg-al (CHEMBL200972) and (e) Flacourtoside F (CHEMBL2036488) were selected for intermolecular interaction studies, structural stability analysis and binding free energy calculations. The best docked poses of these compounds have shown interactions with one or more active site residues His41, Asn142, Gly143, Cys145, His163 and Glu166 of M^pro^. Further, the dynamic stability of each protein-ligand complex was estimated using 100 ns MD simulation, where CHEMBL200972 has shown maximum dynamic stability with minimum fluctuation over the 100 ns MD trajectory. All five compounds’ MM/GBSA binding energies were better compared to the control molecule (X77). Complimenting to the MD simulation results, CHEMBL200972 showed the best ΔG score making it a potential hit for M^pro^. By directly interacting with the substrate binding residues of M^pro^, these five compounds may block the polyprotein processing of SARS-CoV-2. These hits also showed π-π interaction with His41, an essential catalytic residue for M^pro^ that helps create the nucleophilic environment to assist natural substrate binding. Any interaction with His 41 can alter the binding site’s nature, further inhibiting the polyprotein processing. In order to assess the potential of the top five screened compounds, the Computational findings of this study need to be validated in an

## Supporting information

S1 File (DOCX)

S2 File (DOCX)
