# Supplementary material for: Repositioning of anti-dengue compounds against SARS-CoV-2 as viral polyprotein processing inhibitor
Source: PLoS One. 2022 Nov 16;17(11):e0277328. doi: 10.1371/journal.pone.0277328 (PMC9668197; doi:10.1371/journal.pone.0277328)

**Repositioning of Anti-Dengue Compounds Against SARS-CoV-2 as Viral Polyprotein Processing Inhibitor**

Leena H. Bajrai^1,2^, Arwa A. Faizo^1,3^, Areej A. Alkhaldy^1,4^, Vivek Dhar Dwivedi^5,6*^, and Esam I. Azhar^1,3*^

^1^Special Infectious Agents Unit – BSL3, King Fahd Medical Research Center, King Abdulaziz University, Jeddah 21362, Saudi Arabia

^2^Biochemistry Department, Faculty of Sciences, King Abdulaziz University, Jeddah 21362, Saudi Arabia

^3^Department of Medical Laboratory Sciences, Faculty of Applied Medical Sciences, King Abdulaziz University, Jeddah 21362, Saudi Arabia

^4^Clinical Nutrition Department, Faculty of Applied Medical Sciences, King Abdulaziz University, Jeddah, 21589, Saudi Arabia

^5^Center for Bioinformatics, Computational and Systems Biology, Pathfinder Research and Training Foundation, Greater Noida 201308, India.

^6^Bioinformatics Research Division, Quanta Calculus, Greater Noida 201310, India

***Corresponding authors**

EIA; Email: [eazhar@kau.edu.sa](mailto:eazhar@kau.edu.sa)

VDD; Email: [vivekdhar@pathfinderfoundation.co.in](mailto:vivekdhar@pathfinderfoundation.co.in); [vivek_bioinformatics@yahoo.com](mailto:vivek_bioinformatics@yahoo.com)

**Table S1. List of Dengue virus inhibitors collected from DenvInd; PubChem CID, Name, Molecular weight, and Molecular formula**

| **Serial No.** | **PubChem CID** | **Compound Name** | **Molecular Weight** | **Molecular Formula** |
| --- | --- | --- | --- | --- |
|  | 2662 | Celecoxib | 381.4 | C17H14F3N3O2S |
|  | 3220 | Emodin | 270.24 | C15H10O5 |
|  | 5361 | Suramin | 1297.3 | C51H40N6O23S6 |
|  | 9228 | Benzoxazole | 119.12 | C7H5NO |
|  | 37542 | Ribavirin | 244.2 | C8H12N4O5 |
|  | 67145 | 5,5'-Methylenedisalicylic acid | 288.25 | C15H12O6 |
|  | 73201 | 5-Hydroxy-7-methoxy-2-phenylchroman-4-one | 270.28 | C16H14O4 |
|  | 75316 | 2-Amino-4-methylbenzoic acid | 151.16 | C8H9NO2 |
|  | 95276 | 1-Benzyl-1,4-dihydronicotinamide | 214.26 | C13H14N2O |
|  | 122108 | Ribavirin 5'-triphosphate | 484.14 | C8H15N4O14P3 |
|  | 224167 | 4,4'-Sulfonyldibenzoic acid | 306.29 | C14H10O6S |
|  | 225906 | 4-Hydroxy-8-nitroquinoline-3-carboxylic acid | 234.16 | C10H6N2O5 |
|  | 256889 | 3,4-Bis(3-nitrobenzoyl)-1,2,5-oxadiazole 2-oxide | 384.26 | C16H8N4O8 |
|  | 424780 | (4-Cyanophenyl) 4-(diaminomethylideneamino)benzoate | 280.28 | C15H12N4O2 |
|  | 427880 | [4-(Diaminomethylideneamino)phenyl] 4-(diaminomethylideneamino)benzoate | 312.33 | C15H16N6O2 |
|  | 439155 | S-adenosyl-L-homocysteine | 384.41 | C14H20N6O5S |
|  | 442428 | Naringin | 580.5 | C27H32O14 |
|  | 446541 | Mycophenolic acid | 320.3 | C17H20O6 |
|  | 520822 | 4-(Trifluoromethyl)benzene-1,2-diamine | 176.14 | C7H7F3N2 |
|  | 641785 | Cardamonin | 270.28 | C16H14O4 |
|  | 727382 | (5E)-5-[(4-hydroxyphenyl)methylidene]imidazolidine-2,4-dione | 204.18 | C10H8N2O3 |
|  | 986480 | (2R)-3-(furan-2-carbonyl)-4-hydroxy-2-(3-phenoxyphenyl)-1-(pyridin-3-ylmethyl)-2H-pyrrol-5-one | 452.5 | C27H20N2O5 |
|  | 992586 | 2-[4-[(4-Bromophenyl)sulfonylamino]-1-hydroxynaphthalen-2-yl]sulfanylacetic acid | 468.3 | C18H14BrNO5S2 |
|  | 1265824 | N-{4-[(5-chloro-2-methylphenyl)sulfamoyl]phenyl}naphthalene-2-sulfonamide | 487 | C23H19ClN2O4S2 |
|  | 1290536 | 4-({4-[(2-Chlorobenzyl)oxy]-3-methoxybenzyl}amino)benzoic acid | 397.8 | C22H20ClNO4 |
|  | 1311699 | (2R)-2-[4-[(E)-[1-[(2,4-dichlorophenyl)methyl]-2,5-dioxoimidazolidin-4-ylidene]methyl]-2-methoxyphenoxy]propanoic acid | 465.3 | C21H18Cl2N2O6 |
|  | 1339470 | (2E)-N-{4-[(2,6-dimethoxypyrimidin-4-yl)sulfamoyl]phenyl}-3-(naphthalen-1-yl)prop-2-enamide | 490.5 | C25H22N4O5S |
|  | 1355953 | 3-[[4-[(4-Bromophenyl)sulfanylmethyl]benzoyl]amino]benzoic acid | 442.3 | C21H16BrNO3S |
|  | 1376755 | 7-hydroxy-1-N,3-N-diphenylnaphthalene-1,3-disulfonamide | 454.5 | C22H18N2O5S2 |
|  | 1558458 | (4R,5R)-5-(3-hydroxyphenyl)-4-[4-[(2-methylphenyl)methoxy]benzoyl]-1-(pyridin-4-ylmethyl)pyrrolidine-2,3-dione | 506.5 | C31H26N2O5 |
|  | 1608850 | 4-[(3R)-3-(4-bromophenyl)-5-(6-chloro-2-oxo-4-phenyl-1H-quinolin-3-yl)-1,3-dihydropyrazol-2-yl]-4-oxobutanoic acid | 578.8 | C28H21BrClN3O4 |
|  | 1652814 | [(4-{[(2,5-Dimethoxyphenyl)sulfonyl]amino}-1-hydroxy-2-naphthyl)thio]acetic acid | 449.5 | C20H19NO7S2 |
|  | 1784865 | (2S)-2-[[2-(3-tert-butyl-5-methyl-7-oxofuro[3,2-g]chromen-6-yl)acetyl]amino]-3-(5-hydroxy-1H-indol-3-yl)propanoic acid | 516.5 | C29H28N2O7 |
|  | 1987843 | 2-[(4-naphthalen-2-yl-1,3-thiazol-2-yl)carbamoyl]benzoic Acid | 374.4 | C21H14N2O3S |
|  | 2133791 | 4-fluoro-N-[4-hydroxy-3-(phenylthio)-1-naphthyl]benzenesulfonamide | 425.5 | C22H16FNO3S2 |
|  | 2185920 | N-[(3S)-3-(furan-2-yl)-3-phenylpropyl]-N-[(4-methoxyphenyl)methyl]furan-3-carboxamide | 415.5 | C26H25NO4 |
|  | 2203542 | 4-[[3-[(3-cyano-4,5,6,7-tetrahydrobenzothiophen-2-yl)carbamoyl]phenyl]sulfonylamino]benzoic Acid | 481.5 | C23H19N3O5S2 |
|  | 2255243 | 3-[2-(4-Anilinophenyl)hydrazinyl]benzenesulfonic acid | 355.4 | C18H17N3O3S |
|  | 2801468 | 4-Chloro-5-methylbenzene-1,2-diamine | 156.61 | C7H9ClN2 |
|  | 2834598 | 3-({[3-(4-Methylphenyl)-1-adamantyl]carbonyl}amino)benzoic acid | 389.5 | C25H27NO3 |
|  | 2939678 | 2-[1-Hydroxy-4-[(3-nitrophenyl)sulfonylamino]naphthalen-2-yl]sulfanylacetic acid | 434.4 | C18H14N2O7S2 |
|  | 2956510 | N-(3-benzylsulfanyl-4-hydroxynaphthalen-1-yl)-3-nitrobenzenesulfonamide | 466.5 | C23H18N2O5S2 |
|  | 3378440 | 1,8-Dihydroxy-4,5-dinitroanthraquinone | 330.21 | C14H6N2O8 |
|  | 3550295 | 2-(2,4-dioxopyrimidin-1-yl)-N-(4-hydroxy-3-nitrophenyl)acetamide | 306.23 | C12H10N4O6 |
|  | 4148341 | N-(3-(benzo[d]thiazol-2-ylthio)-4-hydroxynaphthalen-1-yl)-3-nitrobenzenesulfonamide | 509.6 | C23H15N3O5S3 |
|  | 4536021 | N-(3-chloro-4-hydroxynaphthalen-1-yl)-4-fluorobenzenesulfonamide | 351.8 | C16H11ClFNO3S |
|  | 4623454 | 2-(Carbamoylamino)-3-(3,4-dihydroxyphenyl)propanoic acid | 240.21 | C10H12N2O5 |
|  | 5036900 | alpha-Aminoorcein | 362.4 | C21H18N2O4 |
|  | 5280343 | Quercetin | 302.23 | C15H10O7 |
|  | 5280459 | Quercitrin | 448.4 | C21H20O11 |
|  | 5280804 | Isoquercitrin | 464.4 | C21H20O12 |
|  | 5280863 | Kaempferol | 286.24 | C15H10O6 |
|  | 5281599 | Agathisflavone | 538.5 | C30H18O10 |
|  | 5281605 | Baicalein | 270.24 | C15H10O5 |
|  | 5281672 | Myricetin | 318.23 | C15H10O8 |
|  | 5281708 | Daidzein | 254.24 | C15H10O4 |
|  | 5281767 | (1E,4Z,6E)-5-hydroxy-1,7-bis(4-hydroxy-3-methoxyphenyl)hepta-1,4,6-trien-3-one | 368.4 | C21H20O6 |
|  | 5429041 | 5-[2,5-dimethyl-3-[(Z)-(phenylcarbamoylhydrazinylidene)methyl]pyrrol-1-yl]benzene-1,3-dicarboxylic acid | 420.4 | C22H20N4O5 |
|  | 5498424 | 2-[2-[(Z)-[[2-[2-[3-(trifluoromethyl)anilino]-1,3-thiazol-4-yl]acetyl]hydrazinylidene]methyl]phenoxy]acetic acid | 478.4 | C21H17F3N4O4S |
|  | 5841996 | 6-[(1-benzyl-1H-indol-3-yl)methylene]-2-(benzylsulfanyl)-5-imino-5,6-dihydro-7H-[1,3,4]thiadiazolo[3,2-a]pyrimidin-7-one | 507.6 | C28H21N5OS2 |
|  | 6150272 | 2-[(Z)-[[4-(naphthalen-2-yloxymethyl)benzoyl]hydrazinylidene]methyl]benzoic acid | 424.4 | C26H20N2O4 |
|  | 6365051 | ({[(Diaminomethylidene)amino]amino}(4-methylphenyl)methyl)phosphinic acid | 241.21 | C9H14N4O2P+ |
|  | 6439576 | Pyracrenic acid | 618.8 | C39H54O6 |
|  | 6857723 | Bz-Nle-Lys-Arg-Arg-H | 659.8 | C31H53N11O5 |
|  | 6906548 | 2-[(E)-[[2-(2-furyl)quinoline-4-carbonyl]hydrazono]methyl]benzoic acid | 385.4 | C22H15N3O4 |
|  | 10406595 | (L)-Methionyl-(L)-prolyl-P-nitroanilide | 366.4 | C16H22N4O4S |
|  | 11628682 | 1-[(1R,2R,4S,5S)-5-carbamimidamido-2,4-bis(2,4-dicarbamimidamidophenoxy)cyclohexyl]guanidine | 610.7 | C24H38N18O2 |
|  | 11635300 | Bz-Lys-Arg-Arg-H | 546.7 | C25H42N10O4 |
|  | 11974567 | Bz-D-Nle-Lys-Arg-Arg-H | 659.8 | C31H53N11O5 |
|  | 11974635 | Bz-Ala-Lys-Arg-Arg-H | 617.7 | C28H47N11O5 |
|  | 11974636 | Bz-Nle-Lys-Phe-Arg-H | 650.8 | C34H50N8O5 |
|  | 11974637 | Bz-Phe-Lys-Arg-Arg-H | 693.8 | C34H51N11O5 |
|  | 11974638 | Bz-Nle-Lys-Arg-Phg-H | 636.8 | C33H48N8O5 |
|  | 11974730 | Bz-Nle-Lys-Arg-(p-CN)Phe-H | 675.8 | C35H49N9O5 |
|  | 11974732 | Bz-Nle-Lys-Arg-Trp-H | 689.8 | C36H51N9O5 |
|  | 11974734 | Bz-Nle-Lys-Lys-Arg-H | 631.8 | C31H53N9O5 |
|  | 11974839 | Bz-Nle-Lys-N-Me-Arg-Arg-H | 673.9 | C32H55N11O5 |
|  | 11974840 | Bz-N-Me-Nle-Lys-Arg-Arg-H | 673.9 | C32H55N11O5 |
|  | 11974841 | Bz-Nle-D-Lys-Arg-Arg-H | 659.8 | C31H53N11O5 |
|  | 11974843 | Bz-Nle-Ala-Arg-Arg-H | 602.7 | C28H46N10O5 |
|  | 11974958 | Bz-Nle-Lys-Arg-Phe-H | 650.8 | C34H50N8O5 |
|  | 11974959 | Bz-Nle-Phe-Arg-Arg-H | 678.8 | C34H50N10O5 |
|  | 11974960 | Bz-Nle-Lys-Arg-(p-Ph)Phe-H | 726.9 | C40H54N8O5 |
|  | 11974962 | Bz-Nle-Lys-Arg-Lys-H | 631.8 | C31H53N9O5 |
|  | 11975081 | Bz-Nle-Lys-Arg-(p-guanidinyl)Phe-H | 707.9 | C35H53N11O5 |
|  | 13555030 | ethyl 4-[[[(E)-benzylideneamino]-phenylcarbamoyl]amino]benzoate | 387.4 | C23H21N3O3 |
|  | 16130513 | Protegrin-1 (RGGRLCYCRRRFCVCVGR-NH2) | 2155.6 | C88H147N37O19S4 |
|  | 19578264 | 2-[4-bromo-2-[(E)-[(3E)-3-[[5-bromo-2-(carboxymethoxy)phenyl]methylidene]-2-oxocyclopentylidene]methyl]phenoxy]acetic acid | 566.2 | C23H18Br2O7 |
|  | 21672233 | [(2R,3S,4S,5R,6S)-3,4,5-trihydroxy-6-(4-hydroxyphenoxy)oxan-2-yl]methyl benzoate | 376.4 | C19H20O8 |
|  | 24320234 | N-(3-(benzylthio)-4-hydroxynaphthalen-1-yl)-4-chlorobenzenesulfonamide | 456 | C23H18ClNO3S2 |
|  | 44406112 | Bz-Arg-Arg-H | 418.5 | C19H30N8O3 |
|  | 44406113 | Bz-Nle-Lys-Arg-(p-Me)Phe-H | 664.8 | C35H52N8O5 |
|  | 44577154 | Itoside H | 526.5 | C27H26O11 |
|  | 44584916 | H-Asn-Gly-Val-Cys(1)-Cys(2)-Gly-Tyr-Lys-Leu-Cys(2)-His-Hyp-Cys(1)-OH | 1408.7 | C57H85N17O17S4 |
|  | 46231130 | 3-[(1R,7R,10S,13S,16S,19S,25S,28S,31R,34S,37S,40S,46S,52S,55S,58R,64S,67S,70S,73S,76S,79S,85R,90S,93S)-90-amino-10,13,37,40,76,79-hexakis(3-carbamimidamidopropyl)-67-(2-carboxyethyl)-34,93-bis[(1R)-1-hydroxyethyl]-16,64,73-tris(hydroxymethyl)-19-(1H-indol-3-ylmethyl)-46-(2-methylpropyl)-2,5,8,11,14,17,20,26,29,32,35,38,41,44,47,53,56,59,62,65,68,71,74,77,80,83,86,88,91,94-triacontaoxo-28,55-di(propan-2-yl)-7,31,58,85-tetrakis(sulfanylmethyl)-3,6,9,12,15,18,21,27,30,33,36,39,42,45,48,54,57,60,63,66,69,72,75,78,81,84,87,92,95-nonacosazatetracyclo[93.3.0.021,25.048,52]octanonacontan-70-yl]propanoic acid | 3202.6 | C129H213N49O39S4 |
|  | 46231132 | 3-[(1R,4S,7S,13S,19S,22S,25S,28R,31S,34S,40S,43S,46S,49S,52R,58S,64S,67S,72R,78S,81S,84S,87S,90S,93S)-67-amino-19,22,46,49,78,81-hexakis(3-carbamimidamidopropyl)-90-(2-carboxyethyl)-25,64-bis[(1R)-1-hydroxyethyl]-43,84,93-tris(hydroxymethyl)-40-(1H-indol-3-ylmethyl)-13-(2-methylpropyl)-3,6,12,15,18,21,24,27,30,33,39,42,45,48,51,54,57,63,66,69,71,74,77,80,83,86,89,92,95,98-triacontaoxo-4,31-di(propan-2-yl)-a,1a,4a,5a-tetrathia-2,5,11,14,17,20,23,26,29,32,38,41,44,47,50,53,56,62,65,70,73,76,79,82,85,88,91,94,97-nonacosazahexacyclo[70.26.4.428,52.07,11.034,38.058,62]hexahectan-87-yl]propanoic acid | 3198.6 | C129H209N49O39S4 |
|  | 46231133 | 3-[(1R,4S,7S,13S,16S,19S,22S,25R,31S,37S,40S,45R,51S,54S,57S,60S,63S,66S,72R,75S,78S,84S,90S,93S,96S)-40-amino-19,22,51,54,90,93-hexakis(3-carbamimidamidopropyl)-63-(2-carboxyethyl)-37,96-bis[(1R)-1-hydroxyethyl]-16,57,66-tris(hydroxymethyl)-13-(1H-indol-3-ylmethyl)-84-(2-methylpropyl)-3,6,12,15,18,21,24,27,30,36,39,42,44,47,50,53,56,59,62,65,68,71,74,77,83,86,89,92,95,98-triacontaoxo-4,75-di(propan-2-yl)-a,1a,4a,5a-tetrathia-2,5,11,14,17,20,23,26,29,35,38,43,46,49,52,55,58,61,64,67,70,73,76,82,85,88,91,94,97-nonacosazahexacyclo[70.26.4.425,45.07,11.031,35.078,82]hexahectan-60-yl]propanoic acid | 3198.6 | C129H209N49O39S4 |
|  | 46233358 | 2-(1-Benzyl-5-methoxy-2,3-dihydroindol-3-yl)acetic acid | 297.3 | C18H19NO3 |
|  | 46233395 | 4-(4-(4-Methoxybenzylidene)-2,3-dioxopyrrolidin-1-yl)butanoic acid | 303.31 | C16H17NO5 |
|  | 46233561 | (4E,5R)-4-[(2,5-dimethylphenyl)-hydroxymethylidene]-5-(4-hydroxyphenyl)-1-(2-phenylethyl)pyrrolidine-2,3-dione | 427.5 | C27H25NO4 |
|  | 46370413 | 3,6-Bis(2,6-dimethylphenyl)-2,3-dihydro-2-thioxo-5-(4-oxo-7-chloro-4H-pyrido[1,2-a]pyrimidine-2-ylmethylthio)thiazolo[4,5-d]pyrimidine-7(6H)-one | 618.2 | C30H24ClN5O2S3 |
|  | 46898022 | n-(4-Aminosulfonylphenyl)-2-morpholino-9h-purin-6-amine | 375.4 | C15H17N7O3S |
|  | 49799036 | N-(4-Aminosulfonylphenyl)-2-(4-(2-hydroxyethyl)piperazin-1-yl)-9H-purin-6-amine | 418.5 | C17H22N8O3S |
|  | 49799133 | 4-(6-Anilino-9H-purine-2-yl)piperazine-1-ethanol | 339.4 | C17H21N7O |
|  | 49848629 | ethyl 3,5-bis[(E)-(1H-benzimidazol-2-ylhydrazinylidene)methyl]benzoate | 466.5 | C25H22N8O2 |
|  | 50800463 | 4-[3-Acetyl-5-(2-phenylquinoline-4-yl)-2,3-dihydro-1,3,4-oxadiazole-2-yl]benzoic acid | 437.4 | C26H19N3O4 |
|  | 56834067 | rac-(6E)-5-(5,7-dihydroxy-4-oxo-2-phenyl-3,4-dihydro-2H-chromen-6-yl)-7-phenylhept-6-enoic acid | 458.5 | C28H26O6 |
|  | 56834069 | (+-)-chartaceone C | 660.7 | C41H40O8 |
|  | 56834070 | (+-)-chartaceone D | 660.7 | C41H40O8 |
|  | 56834169 | (+-)-chartaceone E | 660.7 | C41H40O8 |
|  | 56834170 | (+-)-chartaceone F | 660.7 | C41H40O8 |
|  | 56834171 | chartaceone A1 | 458.5 | C28H26O6 |
|  | 56834172 | chartaceone A2 | 458.5 | C28H26O6 |
|  | 56834173 | Chartaceone A3 | 458.5 | C28H26O6 |
|  | 56834283 | Chartaceone A4 | 458.5 | C28H26O6 |
|  | 57409245 | Flacourtoside C | 670.6 | C33H34O15 |
|  | 57409246 | Flacourtoside D | 698.6 | C34H34O16 |
|  | 57409247 | Flacourtoside E | 680.6 | C34H32O15 |
|  | 57409350 | Flacourtoside F | 784.7 | C41H36O16 |
|  | 60165190 | Scolochinenoside D | 646.6 | C34H30O13 |
|  | 60194816 | N-[(E)-(3-hydroxyphenyl)methylideneamino]-2-[(E)-[(4-sulfamoylphenyl)hydrazinylidene]methyl]quinoline-4-carboxamide | 488.5 | C24H20N6O4S |
|  | 60195084 | 3-[(2E)-2-[[4-(3-phenylpropylcarbamoyl)quinolin-2-yl]methylidene]hydrazinyl]benzoic acid | 452.5 | C27H24N4O3 |
|  | 60195085 | 3-[(2E)-2-[[4-(4-phenylbutylcarbamoyl)quinolin-2-yl]methylidene]hydrazinyl]benzoic acid | 466.5 | C28H26N4O3 |
|  | 60196143 | 2-[(E)-[(3-chlorophenyl)hydrazinylidene]methyl]-N-[(E)-3-phenylpropylideneamino]quinoline-4-carboxamide | 455.9 | C26H22ClN5O |
|  | 70683874 | Xylosmin | 680.6 | C34H32O15 |
|  | 71449745 | 2-[(E)-[(3-nitrophenyl)hydrazinylidene]methyl]-N-[(Z)-[(E)-3-phenylprop-2-enylidene]amino]quinoline-4-carboxamide | 464.5 | C26H20N6O3 |
|  | 71451559 | N-[(Z)-2-phenylethylideneamino]-2-[(E)-[[3-(trifluoromethyl)phenyl]hydrazinylidene]methyl]quinoline-4-carboxamide | 475.5 | C26H20F3N5O |
|  | 71451581 | H-Cys(1)-Gly-Tyr-Lys-Gly-Cys(1)-OH | 627.7 | C25H37N7O8S2 |
|  | 71453400 | 2-[(E)-[(3-bromophenyl)hydrazinylidene]methyl]-N-[(Z)-2-phenylethylideneamino]quinoline-4-carboxamide | 486.4 | C25H20BrN5O |
|  | 71453420 | H-Cys(1)-Gly-D-Tyr-Lys-Leu-Cys(1)-OH | 683.8 | C29H45N7O8S2 |
|  | 71455097 | N-[(Z)-3-phenylpropylideneamino]-2-[(E)-[(4-sulfamoylphenyl)hydrazinylidene]methyl]quinoline-4-carboxamide | 500.6 | C26H24N6O3S |
|  | 71455098 | 2-[(E)-[(3-bromophenyl)hydrazinylidene]methyl]-N-[(Z)-3-phenylpropylideneamino]quinoline-4-carboxamide | 500.4 | C26H22BrN5O |
|  | 71455120 | H-Cys-Ala-Gly-Lys-Arg-Lys-Ser-Gly-OH | 805.9 | C31H59N13O10S |
|  | 71455121 | H-Cys(1)-Gly-Tyr-Lys-Arg-Cys(1)-OH | 726.9 | C29H46N10O8S2 |
|  | 71456909 | 2-[(E)-[(3-chlorophenyl)hydrazinylidene]methyl]-N-[(Z)-2-phenylethylideneamino]quinoline-4-carboxamide | 441.9 | C25H20ClN5O |
|  | 71458760 | 2-[(E)-[(3-chlorophenyl)hydrazinylidene]methyl]-N-[(Z)-[(E)-3-phenylprop-2-enylidene]amino]quinoline-4-carboxamide | 453.9 | C26H20ClN5O |
|  | 71458788 | cyclo[Ala-Gly-D-Lys-Arg-Lys-Ser-Gly-D-Cys] | 787.9 | C31H57N13O9S |
|  | 71458789 | cyclo[Ala-Gly-D-Lys-Arg-Lys-Ser-Gly] | 684.8 | C28H52N12O8 |
|  | 71460599 | H-Cys(1)-Gly-D-Lys-Arg-Lys-Leu-Cys(1)-OH | 805 | C32H60N12O8S2 |
|  | 71460600 | H-Cys(1)-Gly-D-Lys-Arg-Arg-Cys(1)-OH | 719.9 | C26H49N13O7S2 |
|  | 71462287 | N-[(Z)-[(E)-3-phenylprop-2-enylidene]amino]-2-[(E)-[(4-sulfamoylphenyl)hydrazinylidene]methyl]quinoline-4-carboxamide | 498.6 | C26H22N6O3S |
|  | 71462305 | H-Cys(1)-Gly-D-Lys-Arg-Lys-Ser-Cys(1)-OH | 778.9 | C29H54N12O9S2 |
|  | 71462306 | H-Cys(1)-Gly-Gly-Lys-Leu-Cys(1)-OH | 577.7 | C22H39N7O7S2 |
|  | 71521871 | 2-[1-[5-(4-Methoxyphenyl)-1,3,4-oxadiazol-2-yl]-2-phenylethyl]-1,2-benzothiazol-3-one | 429.5 | C24H19N3O3S |
|  | 72710920 | N-[(2S)-1-[[(2S)-6-amino-1-[[(2S)-1-amino-1-oxohexan-2-yl]amino]-1-oxohexan-2-yl]amino]-5-(diaminomethylideneamino)-1-oxopentan-2-yl]-4-[(Z)-(3-cyclohexyl-4-oxo-2-sulfanylidene-1,3-thiazolidin-5-ylidene)methyl]benzamide | 744 | C35H53N9O5S2 |
|  | 72710921 | N-[(2S)-1-[[(2S)-6-amino-1-[[(2S)-1-amino-1-oxohexan-2-yl]amino]-1-oxohexan-2-yl]amino]-5-(diaminomethylideneamino)-1-oxopentan-2-yl]-4-[(Z)-(3-cyclohexyl-2,4-dioxo-1,3-thiazolidin-5-ylidene)methyl]benzamide | 727.9 | C35H53N9O6S |
|  | 72710923 | methyl 2-[(5Z)-5-[[4-[[(2S)-1-[[(2S)-6-amino-1-[[(2S)-1-amino-1-oxohexan-2-yl]amino]-1-oxohexan-2-yl]amino]-5-(diaminomethylideneamino)-1-oxopentan-2-yl]carbamoyl]phenyl]methylidene]-4-oxo-2-sulfanylidene-1,3-thiazolidin-3-yl]hexanoate | 790 | C36H55N9O7S2 |
|  | 72711375 | 2-[(5Z)-5-[[4-[[(2S)-1-[[(2S)-6-amino-1-[[(2S)-1-amino-1-oxohexan-2-yl]amino]-1-oxohexan-2-yl]amino]-5-(diaminomethylideneamino)-1-oxopentan-2-yl]carbamoyl]phenyl]methylidene]-2,4-dioxo-1,3-thiazolidin-3-yl]ethyl acetate | 731.9 | C33H49N9O8S |
|  | 72711810 | N-[(2S)-1-[[(2S)-6-amino-1-[[(2S)-1-amino-1-oxohexan-2-yl]amino]-1-oxohexan-2-yl]amino]-5-(diaminomethylideneamino)-1-oxopentan-2-yl]-4-[(Z)-(3-benzyl-4-oxo-2-sulfanylidene-1,3-thiazolidin-5-ylidene)methyl]benzamide | 752 | C36H49N9O5S2 |
|  | 72711811 | N-[(2S)-1-[[(2S)-6-amino-1-[[(2S)-1-amino-1-oxohexan-2-yl]amino]-1-oxohexan-2-yl]amino]-5-(diaminomethylideneamino)-1-oxopentan-2-yl]-4-[(Z)-(3-benzyl-2,4-dioxo-1,3-thiazolidin-5-ylidene)methyl]benzamide | 735.9 | C36H49N9O6S |
|  | 72711812 | N-[(2S)-1-[[(2S)-6-amino-1-[[(2S)-1-amino-1-oxohexan-2-yl]amino]-1-oxohexan-2-yl]amino]-5-(diaminomethylideneamino)-1-oxopentan-2-yl]-4-[(Z)-[3-[(4-methylphenyl)methyl]-4-oxo-2-sulfanylidene-1,3-thiazolidin-5-ylidene]methyl]benzamide | 766 | C37H51N9O5S2 |
|  | 72711813 | N-[(2S)-1-[[(2S)-6-amino-1-[[(2S)-1-amino-1-oxohexan-2-yl]amino]-1-oxohexan-2-yl]amino]-5-(diaminomethylideneamino)-1-oxopentan-2-yl]-4-[(Z)-[3-[(4-methylphenyl)methyl]-2,4-dioxo-1,3-thiazolidin-5-ylidene]methyl]benzamide | 749.9 | C37H51N9O6S |
|  | 72712026 | N-[(2S)-1-[[(2S)-6-amino-1-[[(2S)-1-amino-1-oxohexan-2-yl]amino]-1-oxohexan-2-yl]amino]-5-(diaminomethylideneamino)-1-oxopentan-2-yl]-4-[(Z)-[3-[(4-methoxyphenyl)methyl]-4-oxo-2-sulfanylidene-1,3-thiazolidin-5-ylidene]methyl]benzamide | 782 | C37H51N9O6S2 |
|  | 72712027 | N-[(2S)-1-[[(2S)-6-amino-1-[[(2S)-1-amino-1-oxohexan-2-yl]amino]-1-oxohexan-2-yl]amino]-5-(diaminomethylideneamino)-1-oxopentan-2-yl]-4-[(Z)-[3-[(4-methoxyphenyl)methyl]-2,4-dioxo-1,3-thiazolidin-5-ylidene]methyl]benzamide | 765.9 | C37H51N9O7S |
|  | 72712028 | N-[(2S)-1-[[(2S)-6-amino-1-[[(2S)-1-amino-1-oxohexan-2-yl]amino]-1-oxohexan-2-yl]amino]-5-(diaminomethylideneamino)-1-oxopentan-2-yl]-4-[(Z)-[3-[(4-fluorophenyl)methyl]-4-oxo-2-sulfanylidene-1,3-thiazolidin-5-ylidene]methyl]benzamide | 770 | C36H48FN9O5S2 |
|  | 72712029 | N-[(2S)-1-[[(2S)-6-amino-1-[[(2S)-1-amino-1-oxohexan-2-yl]amino]-1-oxohexan-2-yl]amino]-5-(diaminomethylideneamino)-1-oxopentan-2-yl]-4-[(Z)-[3-[(4-fluorophenyl)methyl]-2,4-dioxo-1,3-thiazolidin-5-ylidene]methyl]benzamide | 753.9 | C36H48FN9O6S |
|  | 72712239 | N-[(2S)-1-[[(2S)-6-amino-1-[[(2S)-1-amino-1-oxohexan-2-yl]amino]-1-oxohexan-2-yl]amino]-5-(diaminomethylideneamino)-1-oxopentan-2-yl]-4-[(Z)-[3-[(4-chlorophenyl)methyl]-4-oxo-2-sulfanylidene-1,3-thiazolidin-5-ylidene]methyl]benzamide | 786.4 | C36H48ClN9O5S2 |
|  | 72712240 | N-[(2S)-1-[[(2S)-6-amino-1-[[(2S)-1-amino-1-oxohexan-2-yl]amino]-1-oxohexan-2-yl]amino]-5-(diaminomethylideneamino)-1-oxopentan-2-yl]-4-[(Z)-[3-[(4-chlorophenyl)methyl]-2,4-dioxo-1,3-thiazolidin-5-ylidene]methyl]benzamide | 770.3 | C36H48ClN9O6S |
|  | 72712241 | N-[(2S)-1-[[(2S)-6-amino-1-[[(2S)-1-amino-1-oxohexan-2-yl]amino]-1-oxohexan-2-yl]amino]-5-(diaminomethylideneamino)-1-oxopentan-2-yl]-4-[(Z)-[4-oxo-3-(2-phenylethyl)-2-sulfanylidene-1,3-thiazolidin-5-ylidene]methyl]benzamide | 766 | C37H51N9O5S2 |
|  | 72712242 | N-[(2S)-1-[[(2S)-6-amino-1-[[(2S)-1-amino-1-oxohexan-2-yl]amino]-1-oxohexan-2-yl]amino]-5-(diaminomethylideneamino)-1-oxopentan-2-yl]-4-[(Z)-[2,4-dioxo-3-(2-phenylethyl)-1,3-thiazolidin-5-ylidene]methyl]benzamide | 749.9 | C37H51N9O6S |
|  | 72713955 | N-[(2S)-1-[[(2S)-6-amino-1-[[(2S)-1-amino-1-oxohexan-2-yl]amino]-1-oxohexan-2-yl]amino]-5-(diaminomethylideneamino)-1-oxopentan-2-yl]-4-[(Z)-(3-butyl-4-oxo-2-sulfanylidene-1,3-thiazolidin-5-ylidene)methyl]benzamide | 718 | C33H51N9O5S2 |
|  | 72714175 | N-[(2S)-1-[[(2S)-6-amino-1-[[(2S)-1-amino-1-oxohexan-2-yl]amino]-1-oxohexan-2-yl]amino]-5-(diaminomethylideneamino)-1-oxopentan-2-yl]-4-[(Z)-(3-butyl-2,4-dioxo-1,3-thiazolidin-5-ylidene)methyl]benzamide | 701.9 | C33H51N9O6S |
|  | 72714177 | N-[(2S)-1-[[(2S)-6-amino-1-[[(2S)-1-amino-1-oxohexan-2-yl]amino]-1-oxohexan-2-yl]amino]-5-(diaminomethylideneamino)-1-oxopentan-2-yl]-4-[(Z)-(3-cyclopentyl-4-oxo-2-sulfanylidene-1,3-thiazolidin-5-ylidene)methyl]benzamide | 730 | C34H51N9O5S2 |
|  | 72714178 | N-[(2S)-1-[[(2S)-6-amino-1-[[(2S)-1-amino-1-oxohexan-2-yl]amino]-1-oxohexan-2-yl]amino]-5-(diaminomethylideneamino)-1-oxopentan-2-yl]-4-[(Z)-(3-cyclopentyl-2,4-dioxo-1,3-thiazolidin-5-ylidene)methyl]benzamide | 713.9 | C34H51N9O6S |
|  | 72723356 | Boc-Met-Pro-pNA | 466.6 | C21H30N4O6S |
|  | 73346535 | Bz-Arg-Lys-Nle-NH2 | 518.7 | C25H42N8O4 |
|  | 73349552 | (2S)-6-amino-N-[(2S)-1-amino-1-oxohexan-2-yl]-2-[[(2S)-5-(diaminomethylideneamino)-2-[[(E)-3-phenylprop-2-enoyl]amino]pentanoyl]amino]hexanamide | 544.7 | C27H44N8O4 |
|  | 73354076 | N-[(2S)-1-[[(2S)-6-amino-1-[[(2S)-1-amino-1-oxohexan-2-yl]amino]-1-oxohexan-2-yl]amino]-5-(diaminomethylideneamino)-1-oxopentan-2-yl]-4-[(E)-2-cyano-3-(cyclopropylamino)-3-oxoprop-1-enyl]benzamide | 652.8 | C32H48N10O5 |
|  | 73355607 | N-[(2S)-1-[[(2S)-6-amino-1-[[(2S)-1-amino-1-oxohexan-2-yl]amino]-1-oxohexan-2-yl]amino]-5-(diaminomethylideneamino)-1-oxopentan-2-yl]-4-[(E)-3-amino-3-oxoprop-1-enyl]benzamide | 587.7 | C28H45N9O5 |
|  | 73357148 | then-2-oyl-Arg-Lys-Nle-NH2 | 524.7 | C23H40N8O4S |
|  | 101880433 | 3-[1-(4-Fluorobenzyl)-1H-indole-3-yl]-2-cyano-N-[5-(benzylthio)-1,3,4-thiadiazole-2-yl]acrylamide | 525.6 | C28H20FN5OS2 |
|  | 101880434 | 3-[1-(2-Fluorobenzyl)-1H-indole-3-yl]-2-cyano-N-[5-(benzylthio)-1,3,4-thiadiazole-2-yl]acrylamide | 525.6 | C28H20FN5OS2 |
|  | 101880436 | 3-[1-(2-Cyanobenzyl)-1H-indole-3-yl]-2-cyano-N-[5-(benzylthio)-1,3,4-thiadiazole-2-yl]acrylamide | 532.6 | C29H20N6OS2 |
|  | 118716882 | (E)-3-(1-benzylindol-3-yl)-N-(5-benzylsulfanyl-1,3,4-thiadiazol-2-yl)-2-cyanoprop-2-enamide | 507.6 | C28H21N5OS2 |
|  | 118716883 | (E)-3-(1-benzylindol-3-yl)-2-cyano-N-[5-(trifluoromethyl)-1,3,4-thiadiazol-2-yl]prop-2-enamide | 453.4 | C22H14F3N5OS |
|  | 118716884 | (E)-3-(1-benzylindol-3-yl)-2-cyano-N-[5-[(4-fluorophenyl)methylsulfanyl]-1,3,4-thiadiazol-2-yl]prop-2-enamide | 525.6 | C28H20FN5OS2 |
|  | 118716886 | (E)-3-(1-benzylindol-3-yl)-2-cyano-N-[5-[(4-methoxyphenyl)methyl]-1,3,4-thiadiazol-2-yl]prop-2-enamide | 505.6 | C29H23N5O2S |
|  | 118717690 | 5-[(4-chloro-3-cyano-5-methylpyrazol-1-yl)methyl]-N-[1-[2-(diethylamino)ethyl]benzimidazol-2-yl]furan-2-carboxamide | 480 | C24H26ClN7O2 |
|  | 118717691 | 2-amino-4-[[(2S,5R)-5-[4-[(3-chlorophenyl)methylamino]pyrrolo[2,3-d]pyrimidin-7-yl]-3,4-dihydroxyoxolan-2-yl]methylsulfanyl]butanoic acid | 508 | C22H26ClN5O5S |
|  | 118717692 | H-Trp-Tyr-Cys-Trp-NH2 | 655.8 | C34H37N7O5S |
|  | 118717693 | 6-[4-(5,7-Dihydroxy-4-oxochromen-2-yl)phenoxy]-5,7-dihydroxy-3-(4-hydroxyphenyl)chromen-4-one | 538.5 | C30H18O10 |
|  | 118779901 | (5-hydroxypyridin-2-yl)-[(1S,9S)-8-oxa-2,3,4,12-tetrazatricyclo[7.4.0.02,6]trideca-3,5-dien-12-yl]methanone | 301.3 | C14H15N5O3 |
|  | 118797900 | 2,2'-(5-(Thiophen-2-Yl)-1,3-Phenylene)diacetic Acid | 276.31 | C14H12O4S |
|  | 118797902 | 5-(5-(3-Hydroxyprop-1-Yn-1-Yl)thiophen-2-Yl)-4-Methoxy-2-Methyl-N-(Methylsulfonyl)benzamide | 379.5 | C17H17NO5S2 |
|  | 121232415 | 5-[5-(3-Hydroxyprop-1-Yn-1-Yl)thiophen-2-Yl]-2,4-Dimethoxy-N-[(3-Methoxyphenyl)sulfonyl]benzamide | 487.5 | C23H21NO7S2 |
|  | 122178194 | 1-[(2R,4S,5S)-5-[[tert-butyl(dimethyl)silyl]oxymethyl]-4-[5-(6-methoxynaphthalen-2-yl)triazol-1-yl]oxolan-2-yl]-5-methylpyrimidine-2,4-dione | 563.7 | C29H37N5O5Si |
|  | 122178202 | 1-[(2R,4S,5S)-5-[[tert-butyl(dimethyl)silyl]oxymethyl]-4-(5-phenyltriazol-1-yl)oxolan-2-yl]-5-methylpyrimidine-2,4-dione | 483.6 | C24H33N5O4Si |
|  | 122178208 | 1-[(2R,4S,5S)-5-[[tert-butyl(dimethyl)silyl]oxymethyl]-4-(5-cyclohexyltriazol-1-yl)oxolan-2-yl]-5-methylpyrimidine-2,4-dione | 489.7 | C24H39N5O4Si |
|  | 122178209 | 1-[(2R,4S,5S)-5-[[tert-butyl(dimethyl)silyl]oxymethyl]-4-[4-(6-methoxynaphthalen-2-yl)triazol-1-yl]oxolan-2-yl]-5-methylpyrimidine-2,4-dione | 563.7 | C29H37N5O5Si |
|  | 122178213 | 1-[(2R,4S,5S)-5-[[tert-butyl(dimethyl)silyl]oxymethyl]-4-[2-(1H-indol-5-yl)-4-oxo-1,3-thiazolidin-3-yl]oxolan-2-yl]-5-methylpyrimidine-2,4-dione | 556.8 | C27H36N4O5SSi |
|  | 122178215 | 1-[(2R,4S,5S)-4-(2-anthracen-9-yl-4-oxo-1,3-thiazolidin-3-yl)-5-[[tert-butyl(dimethyl)silyl]oxymethyl]oxolan-2-yl]-5-methylpyrimidine-2,4-dione | 617.8 | C33H39N3O5SSi |
|  | 122178221 | N-[(2S,3S,5R)-2-[[tert-butyl(dimethyl)silyl]oxymethyl]-5-(5-methyl-2,4-dioxopyrimidin-1-yl)oxolan-3-yl]-2,2,2-triphenylacetamide | 625.8 | C36H43N3O5Si |
|  | 122178223 | N-[(2S,3S,5R)-2-[[tert-butyl(dimethyl)silyl]oxymethyl]-5-(5-methyl-2,4-dioxopyrimidin-1-yl)oxolan-3-yl]naphthalene-1-carboxamide | 509.7 | C27H35N3O5Si |
|  | 122178230 | N-[(2S,3S,5R)-2-[[tert-butyl(dimethyl)silyl]oxymethyl]-5-(5-methyl-2,4-dioxopyrimidin-1-yl)oxolan-3-yl]-5-(4-fluorophenyl)pentanamide | 533.7 | C27H40FN3O5Si |
|  | 122178231 | N-[(2S,3S,5R)-2-[[tert-butyl(dimethyl)silyl]oxymethyl]-5-(5-methyl-2,4-dioxopyrimidin-1-yl)oxolan-3-yl]naphthalene-2-sulfonamide | 545.7 | C26H35N3O6SSi |
|  | 122178232 | 1-[(2S,3S,5R)-2-[[tert-butyl(dimethyl)silyl]oxymethyl]-5-(5-methyl-2,4-dioxopyrimidin-1-yl)oxolan-3-yl]-3-naphthalen-1-ylurea | 524.7 | C27H36N4O5Si |
|  | 122178233 | 1-(1-adamantyl)-3-[(2S,3S,5R)-2-[[tert-butyl(dimethyl)silyl]oxymethyl]-5-(5-methyl-2,4-dioxopyrimidin-1-yl)oxolan-3-yl]urea | 532.7 | C27H44N4O5Si |
|  | 122178649 | 4-(2-(Isopentylamino)-9H-purine-6-ylamino)-alpha-methylbenzaldehyde | 338.4 | C18H22N6O |
|  | 122178650 | 4-(2-(Methylamino)-9H-purine-6-ylamino)-alpha-methylbenzaldehyde | 282.3 | C14H14N6O |
|  | 122178651 | 4-(2-(2-Hydroxyethylamino)-9H-purine-6-ylamino)-alpha-methylbenzaldehyde | 312.33 | C15H16N6O2 |
|  | 122178652 | 3-(2-(Benzylamino)-9H-purine-6-ylamino)phenol | 332.4 | C18H16N6O |
|  | 122178653 | 3-(2-(Isopentylamino)-9H-purine-6-ylamino)phenol | 312.37 | C16H20N6O |
|  | 122178654 | 3-(2-(Methylamino)-9H-purine-6-ylamino)phenol | 256.26 | C12H12N6O |
|  | 122178655 | 3-(2-(2-(1H-Imidazole-4-yl)ethylamino)-9H-purine-6-ylamino)phenol | 336.35 | C16H16N8O |
|  | 122178656 | 3-[[2-(2-hydroxyethylamino)-7H-purin-6-yl]amino]phenol | 286.29 | C13H14N6O2 |
|  | 122193290 | Bz-Phe(4-MeNH2)-Lys-Phg-NH2 | 558.7 | C31H38N6O4 |
|  | 122193291 | Bz-4-Guanyl-L-Phe-L-Lys-L-phenyl Gly-NH2 | 571.7 | C31H37N7O4 |
|  | 122193292 | Bz-4-Guanidino-L-Phe-L-Lys-L-phenyl Gly-NH2 | 586.7 | C31H38N8O4 |
|  | 122193294 | Bz-Phe(Unk)-Lys-Phg-NH2 | 585.7 | C32H39N7O4 |
|  | 122193299 | N-[(2S)-1-[[(2S)-6-amino-1-[[(1S)-2-amino-2-oxo-1-phenylethyl]amino]-1-oxohexan-2-yl]amino]-3-[3-(aminomethyl)phenyl]-1-oxopropan-2-yl]benzamide | 558.7 | C31H38N6O4 |
|  | 122193300 | Bz-Phe(3-amidino)-Lys-Phg-NH2 | 571.7 | C31H37N7O4 |
|  | 122193301 | N-[(2S)-1-[[(2S)-6-amino-1-[[(1S)-2-amino-2-oxo-1-phenylethyl]amino]-1-oxohexan-2-yl]amino]-3-[3-(diaminomethylideneamino)phenyl]-1-oxopropan-2-yl]benzamide | 586.7 | C31H38N8O4 |
|  | 122193302 | N-[(1S)-2-[[(2S)-6-amino-1-[[(1S)-2-amino-2-oxo-1-phenylethyl]amino]-1-oxohexan-2-yl]amino]-1-[3-(diaminomethylideneamino)phenyl]-2-oxoethyl]benzamide | 572.7 | C30H36N8O4 |
|  | 122193303 | Bz(4-CF3)-Arg-Lys-Phg-NH2 | 606.6 | C28H37F3N8O4 |
|  | 122193304 | Bz(4-CF3)-Phe(4-amidino)-Lys-Phg-NH2 | 639.7 | C32H36F3N7O4 |
|  | 122193305 | Bz(4-CF3)-Phe(3-amidino)-Lys-Phg-NH2 | 639.7 | C32H36F3N7O4 |
|  | 122193306 | Bz(4-CF3)-Phe(4-guanidino)-Lys-Phg-NH2 | 654.7 | C32H37F3N8O4 |
|  | 122193307 | deamino-Phe-Arg-Lys-Phg-NH2 | 566.7 | C29H42N8O4 |
|  | 122193308 | deamino-Phe-Phe(4-amidino)-Lys-Phg-NH2 | 599.7 | C33H41N7O4 |
|  | 122193309 | deamino-Phe-Phe(3-amidino)-Lys-Phg-NH2 | 599.7 | C33H41N7O4 |
|  | 122193488 | deamino-Phe-Phe(4-guanidino)-Lys-Phg-NH2 | 614.7 | C33H42N8O4 |
|  | 122193489 | H-4Abz-Phe(4-amidino)-Lys-Phg-NH2 | 586.7 | C31H38N8O4 |
|  | 122197561 | 4-[3-[(2-Azanyl-4-Chloranyl-Phenyl)carbamoylamino]phenyl]sulfonyloxybenzoic Acid | 461.9 | C20H16ClN3O6S |
|  | 122197562 | 4-[[3-[(2-Azanyl-4-Chloranyl-Phenyl)carbamoylamino]phenyl]sulfonylamino]benzoic Acid | 460.9 | C20H17ClN4O5S |
|  | 127037662 | N-[(2S)-1-[[(2S)-6-amino-1-[[(1R)-2-amino-1-[4-[(4-cyanophenyl)methoxy]phenyl]-2-oxoethyl]amino]-1-oxohexan-2-yl]amino]-5-(diaminomethylideneamino)-1-oxopentan-2-yl]benzamide | 669.8 | C35H43N9O5 |
|  | 127037663 | Bz-Arg-Lys-D-nTyr(Bn(4-Br))-NH2 | 723.7 | C34H43BrN8O5 |
|  | 127037697 | then-2-oyl-Arg-Lys-D-nTyr(Bn)-NH2 | 650.8 | C32H42N8O5S |
|  | 127037698 | then-3-oyl-Arg-Lys-D-nTyr(Bn)-NH2 | 650.8 | C32H42N8O5S |
|  | 127037699 | N-[(2S)-1-[[(2S)-6-amino-1-[[(1R)-2-amino-2-oxo-1-(4-phenylmethoxyphenyl)ethyl]amino]-1-oxohexan-2-yl]amino]-5-(diaminomethylideneamino)-1-oxopentan-2-yl]-1,3-thiazole-4-carboxamide | 651.8 | C31H41N9O5S |
|  | 127037700 | N-[(2S)-1-[[(2S)-6-amino-1-[[(1R)-2-amino-2-oxo-1-(4-phenylmethoxyphenyl)ethyl]amino]-1-oxohexan-2-yl]amino]-5-(diaminomethylideneamino)-1-oxopentan-2-yl]-1,3-thiazole-2-carboxamide | 651.8 | C31H41N9O5S |
|  | 127037749 | N-[(2S)-1-[[(2S)-6-amino-1-[[(1R)-2-amino-2-oxo-1-(4-phenylmethoxyphenyl)ethyl]amino]-1-oxohexan-2-yl]amino]-5-(diaminomethylideneamino)-1-oxopentan-2-yl]-2,3-dihydrothieno[3,4-b][1,4]dioxine-5-carboxamide | 708.8 | C34H44N8O7S |
|  | 127037750 | N-[(2S)-1-[[(2S)-6-amino-1-[[(1R)-2-amino-2-oxo-1-(4-phenylmethoxyphenyl)ethyl]amino]-1-oxohexan-2-yl]amino]-5-(diaminomethylideneamino)-1-oxopentan-2-yl]-5-thiophen-2-ylthiophene-2-carboxamide | 732.9 | C36H44N8O5S2 |
|  | 127037751 | N-[(2S)-1-[[(2S)-6-amino-1-[[(1R)-2-amino-2-oxo-1-(4-phenylmethoxyphenyl)ethyl]amino]-1-oxohexan-2-yl]amino]-5-(diaminomethylideneamino)-1-oxopentan-2-yl]-3-pyrrol-1-ylthiophene-2-carboxamide | 715.9 | C36H45N9O5S |
|  | 127038018 | Bz-Arg-Lys-D-nTyr(Bn(4-F))-NH2 | 662.8 | C34H43FN8O5 |
|  | 127038019 | Bz-Arg-Lys-D-nTyr(Bn(4-Cl))-NH2 | 679.2 | C34H43ClN8O5 |
|  | 127038036 | Bz-Arg-Lys-D-nTyr(Bn(4-NH2))-NH2 | 659.8 | C34H45N9O5 |
|  | 127038037 | Bz-Arg-Lys-D-nTyr(Bn(2-Cl))-NH2 | 679.2 | C34H43ClN8O5 |
|  | 127038392 | Bz-Arg-Lys-D-nTyr(Bn(3-Cl))-NH2 | 679.2 | C34H43ClN8O5 |
|  | 127038393 | Bz-Arg-Lys-D-nTyr(Bn(2,6-diCl))-NH2 | 713.7 | C34H42Cl2N8O5 |
|  | 127038504 | N-[(2S)-1-[[(2S)-6-amino-1-[[(1R)-2-amino-1-[4-[(3-methoxyphenyl)methoxy]phenyl]-2-oxoethyl]amino]-1-oxohexan-2-yl]amino]-5-(diaminomethylideneamino)-1-oxopentan-2-yl]-1,3-thiazole-2-carboxamide | 681.8 | C32H43N9O6S |
|  | 127038505 | N-[(2S)-1-[[(2S)-6-amino-1-[[(1R)-2-amino-1-[4-[(2,6-dichlorophenyl)methoxy]phenyl]-2-oxoethyl]amino]-1-oxohexan-2-yl]amino]-5-(diaminomethylideneamino)-1-oxopentan-2-yl]-1,3-thiazole-2-carboxamide | 720.7 | C31H39Cl2N9O5S |
|  | 127038506 | N-[(2S)-1-[[(2S)-6-amino-1-[[(1R)-2-amino-2-oxo-1-[4-[[4-(trifluoromethyl)phenyl]methoxy]phenyl]ethyl]amino]-1-oxohexan-2-yl]amino]-5-(diaminomethylideneamino)-1-oxopentan-2-yl]thiophene-2-carboxamide | 718.8 | C33H41F3N8O5S |
|  | 127038827 | N-[(2S)-1-[[(2S)-6-amino-1-[[(1R)-2-amino-2-oxo-1-(4-phenylmethoxyphenyl)ethyl]amino]-1-oxohexan-2-yl]amino]-5-(diaminomethylideneamino)-1-oxopentan-2-yl]-1-benzothiophene-2-carboxamide | 700.9 | C36H44N8O5S |
|  | 127038864 | then-2-oyl-Arg-Lys-D-nTyr(Bn(4-F))-NH2 | 668.8 | C32H41FN8O5S |
|  | 127038865 | N-[(2S)-1-[[(2S)-6-amino-1-[[(1R)-2-amino-1-[4-[(4-chlorophenyl)methoxy]phenyl]-2-oxoethyl]amino]-1-oxohexan-2-yl]amino]-5-(diaminomethylideneamino)-1-oxopentan-2-yl]-5-chlorothiophene-2-carboxamide | 719.7 | C32H40Cl2N8O5S |
|  | 127038866 | N-[(2S)-1-[[(2S)-6-amino-1-[[(1R)-2-amino-1-[4-[(4-tert-butylphenyl)methoxy]phenyl]-2-oxoethyl]amino]-1-oxohexan-2-yl]amino]-5-(diaminomethylideneamino)-1-oxopentan-2-yl]-5-chlorothiophene-2-carboxamide | 741.3 | C36H49ClN8O5S |
|  | 127038867 | N-[(2S)-1-[[(2S)-6-amino-1-[[(1R)-2-amino-1-[4-[(4-chlorophenyl)methoxy]phenyl]-2-oxoethyl]amino]-1-oxohexan-2-yl]amino]-5-(diaminomethylideneamino)-1-oxopentan-2-yl]-3-chlorothiophene-2-carboxamide | 719.7 | C32H40Cl2N8O5S |
|  | 127039151 | N-[(2S)-1-[[(2S)-6-amino-1-[[(1R)-2-amino-2-oxo-1-(4-phenylmethoxyphenyl)ethyl]amino]-1-oxohexan-2-yl]amino]-5-(diaminomethylideneamino)-1-oxopentan-2-yl]-4-phenylthiophene-2-carboxamide | 726.9 | C38H46N8O5S |
|  | 127039185 | N-[(2S)-1-[[(2S)-6-amino-1-[[(1R)-2-amino-1-[4-[(4-tert-butylphenyl)methoxy]phenyl]-2-oxoethyl]amino]-1-oxohexan-2-yl]amino]-5-(diaminomethylideneamino)-1-oxopentan-2-yl]-3-chlorothiophene-2-carboxamide | 741.3 | C36H49ClN8O5S |
|  | 127039186 | N-[(2S)-1-[[(2S)-6-amino-1-[[(1R)-2-amino-2-oxo-1-[4-[[4-(trifluoromethyl)phenyl]methoxy]phenyl]ethyl]amino]-1-oxohexan-2-yl]amino]-5-(diaminomethylideneamino)-1-oxopentan-2-yl]thieno[2,3-b]thiophene-5-carboxamide | 774.9 | C35H41F3N8O5S2 |
|  | 127039187 | N-[(2S)-1-[[(2S)-6-amino-1-[[(1R)-2-amino-2-oxo-1-[4-[[4-(trifluoromethyl)phenyl]methoxy]phenyl]ethyl]amino]-1-oxohexan-2-yl]amino]-5-(diaminomethylideneamino)-1-oxopentan-2-yl]-1-benzothiophene-2-carboxamide | 768.8 | C37H43F3N8O5S |
|  | 127039188 | N-[(2S)-1-[[(2S)-6-amino-1-[[(1R)-2-amino-2-oxo-1-[4-[[2-(trifluoromethyl)phenyl]methoxy]phenyl]ethyl]amino]-1-oxohexan-2-yl]amino]-5-(diaminomethylideneamino)-1-oxopentan-2-yl]-1-benzothiophene-2-carboxamide | 768.8 | C37H43F3N8O5S |
|  | 127039442 | N-[(2S)-1-[[(2S)-6-amino-1-[[(1R)-2-amino-2-oxo-1-(4-phenylmethoxyphenyl)ethyl]amino]-1-oxohexan-2-yl]amino]-5-(diaminomethylideneamino)-1-oxopentan-2-yl]-1,3-thiazole-5-carboxamide | 651.8 | C31H41N9O5S |
|  | 127039443 | cHexCO-Arg-Lys-D-nTyr(Bn)-NH2 | 650.8 | C34H50N8O5 |
|  | 127039444 | cPentCO-Arg-Lys-D-nTyr(Bn)-NH2 | 636.8 | C33H48N8O5 |
|  | 127039475 | N-[(2S)-1-[[(2S)-6-amino-1-[[(1R)-2-amino-2-oxo-1-(4-phenylmethoxyphenyl)ethyl]amino]-1-oxohexan-2-yl]amino]-5-(diaminomethylideneamino)-1-oxopentan-2-yl]-5-phenylthiophene-2-carboxamide | 726.9 | C38H46N8O5S |
|  | 127039493 | Bz-Arg-Lys-D-Phg-NH2 | 538.6 | C27H38N8O4 |
|  | 127039494 | Bz-Arg-Lys-nTyr-NH2 | 554.6 | C27H38N8O5 |
|  | 127039520 | N-[(2S)-1-[[(2S)-6-amino-1-[[(1R)-2-amino-1-[4-[(2,6-dichlorophenyl)methoxy]phenyl]-2-oxoethyl]amino]-1-oxohexan-2-yl]amino]-5-(diaminomethylideneamino)-1-oxopentan-2-yl]-1-benzothiophene-2-carboxamide | 769.7 | C36H42Cl2N8O5S |
|  | 127039757 | Bz-Arg-Lys-D-nTyr(Bn(3,4-diCl))-NH2 | 713.7 | C34H42Cl2N8O5 |
|  | 127039758 | Bz-Arg-Lys-D-nTyr(Bn(3-OMe))-NH2 | 674.8 | C35H46N8O6 |
|  | 127039770 | cBuCO-Arg-Lys-D-nTyr(Bn)-NH2 | 622.8 | C32H46N8O5 |
|  | 127039816 | N-[(2S)-1-[[(2S)-6-amino-1-[[(1R)-2-amino-2-oxo-1-[4-[[4-(trifluoromethyl)phenyl]methoxy]phenyl]ethyl]amino]-1-oxohexan-2-yl]amino]-5-(diaminomethylideneamino)-1-oxopentan-2-yl]-1,3-thiazole-2-carboxamide | 719.8 | C32H40F3N9O5S |
|  | 127039817 | then-2-oyl-Arg-Lys-D-nTyr(Bn(3-OMe))-NH2 | 680.8 | C33H44N8O6S |
|  | 127039818 | then-2-oyl-Arg-Lys-D-nTyr(Bn(4-Br))-NH2 | 729.7 | C32H41BrN8O5S |
|  | 127040105 | Bz-Arg-Lys-D-nTyr(Bn(3-Me))-NH2 | 658.8 | C35H46N8O5 |
|  | 127040106 | Bz-Arg-Lys-D-nTyr(Bn(4-tBu))-NH2 | 700.9 | C38H52N8O5 |
|  | 127040128 | cPrCO-Arg-Lys-D-nTyr(Bn)-NH2 | 608.7 | C31H44N8O5 |
|  | 127040129 | N-[(2S)-1-[[(2S)-6-amino-1-[[(1R)-2-amino-2-oxo-1-(4-phenylmethoxyphenyl)ethyl]amino]-1-oxohexan-2-yl]amino]-5-(diaminomethylideneamino)-1-oxopentan-2-yl]-3-chlorothiophene-2-carboxamide | 685.2 | C32H41ClN8O5S |
|  | 127040160 | then-2-oyl-Arg-Lys-D-nTyr(Bn(3,4-diCl))-NH2 | 719.7 | C32H40Cl2N8O5S |
|  | 127040161 | N-[(2S)-1-[[(2S)-6-amino-1-[[(1R)-2-amino-1-[4-[2-(4-bromophenyl)-2-oxoethoxy]phenyl]-2-oxoethyl]amino]-1-oxohexan-2-yl]amino]-5-(diaminomethylideneamino)-1-oxopentan-2-yl]thiophene-2-carboxamide | 757.7 | C33H41BrN8O6S |
|  | 127040162 | N-[(2S)-1-[[(2S)-6-amino-1-[[(1R)-2-amino-1-[4-[(4-tert-butylphenyl)methoxy]phenyl]-2-oxoethyl]amino]-1-oxohexan-2-yl]amino]-5-(diaminomethylideneamino)-1-oxopentan-2-yl]thieno[3,2-b]thiophene-5-carboxamide | 763 | C38H50N8O5S2 |
|  | 127040163 | N-[(2S)-1-[[(2S)-6-amino-1-[[(1R)-2-amino-1-[4-[(2,6-dichlorophenyl)methoxy]phenyl]-2-oxoethyl]amino]-1-oxohexan-2-yl]amino]-5-(diaminomethylideneamino)-1-oxopentan-2-yl]thieno[3,2-b]thiophene-5-carboxamide | 775.8 | C34H40Cl2N8O5S2 |
|  | 127040493 | N-[(2S)-1-[[(2S)-6-amino-1-[[(1R)-2-amino-2-oxo-1-(4-phenylmethoxyphenyl)ethyl]amino]-1-oxohexan-2-yl]amino]-5-(diaminomethylideneamino)-1-oxopentan-2-yl]-5-chlorothiophene-2-carboxamide | 685.2 | C32H41ClN8O5S |
|  | 127040494 | N-[(2S)-1-[[(2S)-6-amino-1-[[(1R)-2-amino-2-oxo-1-(4-phenylmethoxyphenyl)ethyl]amino]-1-oxohexan-2-yl]amino]-5-(diaminomethylideneamino)-1-oxopentan-2-yl]-5-methylthiophene-2-carboxamide | 664.8 | C33H44N8O5S |
|  | 127040497 | Bz(4-F)-Arg-Lys-D-nTyr(Bn)-NH2 | 662.8 | C34H43FN8O5 |
|  | 127040498 | Bz(3-F)-Arg-Lys-D-nTyr(Bn)-NH2 | 662.8 | C34H43FN8O5 |
|  | 127040511 | N-[(2S)-1-[[(2S)-6-amino-1-[[(1R)-2-amino-1-[4-[(4-chlorophenyl)methoxy]phenyl]-2-oxoethyl]amino]-1-oxohexan-2-yl]amino]-5-(diaminomethylideneamino)-1-oxopentan-2-yl]thieno[3,2-b]thiophene-5-carboxamide | 741.3 | C34H41ClN8O5S2 |
|  | 127040512 | N-[(2S)-1-[[(2S)-6-amino-1-[[(1R)-2-amino-2-oxo-1-[4-[[2-(trifluoromethyl)phenyl]methoxy]phenyl]ethyl]amino]-1-oxohexan-2-yl]amino]-5-(diaminomethylideneamino)-1-oxopentan-2-yl]thieno[3,2-b]thiophene-5-carboxamide | 774.9 | C35H41F3N8O5S2 |
|  | 127040513 | N-[(2S)-1-[[(2S)-6-amino-1-[[(1R)-2-amino-1-[4-[(3-methoxyphenyl)methoxy]phenyl]-2-oxoethyl]amino]-1-oxohexan-2-yl]amino]-5-(diaminomethylideneamino)-1-oxopentan-2-yl]-5-thiophen-2-ylthiophene-2-carboxamide | 762.9 | C37H46N8O6S2 |
|  | 127040514 | Unk-Arg-Lys-D-nTyr(Bn(3,4-diCl))-NH2 | 801.8 | C36H42Cl2N8O5S2 |
|  | 127040762 | N-[(2S)-1-[[(2S)-6-amino-1-[[(1R)-2-amino-2-oxo-1-[4-[[4-(trifluoromethyl)phenyl]methoxy]phenyl]ethyl]amino]-1-oxohexan-2-yl]amino]-5-(diaminomethylideneamino)-1-oxopentan-2-yl]benzamide | 712.8 | C35H43F3N8O5 |
|  | 127040763 | N-[(2S)-1-[[(2S)-6-amino-1-[[(1R)-2-amino-2-oxo-1-[4-[[4-(trifluoromethoxy)phenyl]methoxy]phenyl]ethyl]amino]-1-oxohexan-2-yl]amino]-5-(diaminomethylideneamino)-1-oxopentan-2-yl]benzamide | 728.8 | C35H43F3N8O6 |
|  | 127040805 | Bz(2-F)-Arg-Lys-D-nTyr(Bn)-NH2 | 662.8 | C34H43FN8O5 |
|  | 127040806 | Bz(2-CF3)-Arg-Lys-D-nTyr(Bn)-NH2 | 712.8 | C35H43F3N8O5 |
|  | 127040811 | N-[(2S)-1-[[(2S)-6-amino-1-[[(1R)-2-amino-2-oxo-1-(4-phenylmethoxyphenyl)ethyl]amino]-1-oxohexan-2-yl]amino]-5-(diaminomethylideneamino)-1-oxopentan-2-yl]-3-formylbenzamide | 672.8 | C35H44N8O6 |
|  | 127040812 | N-[(2S)-1-[[(2S)-6-amino-1-[[(1R)-2-amino-2-oxo-1-(4-phenylmethoxyphenyl)ethyl]amino]-1-oxohexan-2-yl]amino]-5-(diaminomethylideneamino)-1-oxopentan-2-yl]-4-formylbenzamide | 672.8 | C35H44N8O6 |
|  | 127040813 | 4-acetyl-N-[(2S)-1-[[(2S)-6-amino-1-[[(1R)-2-amino-2-oxo-1-(4-phenylmethoxyphenyl)ethyl]amino]-1-oxohexan-2-yl]amino]-5-(diaminomethylideneamino)-1-oxopentan-2-yl]benzamide | 686.8 | C36H46N8O6 |
|  | 127040814 | (2S)-6-amino-N-[(1R)-2-amino-2-oxo-1-(4-phenylmethoxyphenyl)ethyl]-2-[[(2S)-5-(diaminomethylideneamino)-2-[[(E)-3-phenylprop-2-enoyl]amino]pentanoyl]amino]hexanamide | 670.8 | C36H46N8O5 |
|  | 127040817 | (2S)-6-amino-N-[(1R)-2-amino-2-oxo-1-(4-phenylmethoxyphenyl)ethyl]-2-[[(2S)-2-[[(E)-3-(4-chlorophenyl)prop-2-enoyl]amino]-5-(diaminomethylideneamino)pentanoyl]amino]hexanamide | 705.2 | C36H45ClN8O5 |
|  | 127040818 | deamino-Phe-Arg-Lys-D-nTyr(Bn)-NH2 | 672.8 | C36H48N8O5 |
|  | 127040819 | N-[(2S)-1-[[(2S)-6-amino-1-[[(1R)-2-amino-2-oxo-1-(4-phenylmethoxyphenyl)ethyl]amino]-1-oxohexan-2-yl]amino]-5-(diaminomethylideneamino)-1-oxopentan-2-yl]thieno[2,3-b]thiophene-5-carboxamide | 706.9 | C34H42N8O5S2 |
|  | 127040820 | N-[(2S)-1-[[(2S)-6-amino-1-[[(1R)-2-amino-1-[4-[(2,6-dichlorophenyl)methoxy]phenyl]-2-oxoethyl]amino]-1-oxohexan-2-yl]amino]-5-(diaminomethylideneamino)-1-oxopentan-2-yl]-4-phenylthiophene-2-carboxamide | 795.8 | C38H44Cl2N8O5S |
|  | 127041096 | Bz-Arg-Lys-D-nTyr(Bn(2-Br))-NH2 | 723.7 | C34H43BrN8O5 |
|  | 127041097 | N-[(2S)-1-[[(2S)-6-amino-1-[[(1R)-2-amino-2-oxo-1-[4-[[2-(trifluoromethyl)phenyl]methoxy]phenyl]ethyl]amino]-1-oxohexan-2-yl]amino]-5-(diaminomethylideneamino)-1-oxopentan-2-yl]benzamide | 712.8 | C35H43F3N8O5 |
|  | 127041151 | N-[(2S)-1-[[(2S)-6-amino-1-[[(1R)-2-amino-2-oxo-1-(4-phenylmethoxyphenyl)ethyl]amino]-1-oxohexan-2-yl]amino]-5-(diaminomethylideneamino)-1-oxopentan-2-yl]thieno[3,2-b]thiophene-5-carboxamide | 706.9 | C34H42N8O5S2 |
|  | 127041429 | Bz-Arg-Lys-D-nTyr(naphth-2-ylmethyl)-NH2 | 694.8 | C38H46N8O5 |
|  | 127041430 | N-[(2S)-1-[[(2S)-6-amino-1-[[(1R)-2-amino-2-oxo-1-(4-phenacyloxyphenyl)ethyl]amino]-1-oxohexan-2-yl]amino]-5-(diaminomethylideneamino)-1-oxopentan-2-yl]benzamide | 672.8 | C35H44N8O6 |
|  | 127041431 | nicotinoyl-Arg-Lys-D-nTyr(Bn)-NH2 | 645.8 | C33H43N9O5 |
|  | 127041432 | fur-2-oyl-Arg-Lys-D-nTyr(Bn)-NH2 | 634.7 | C32H42N8O6 |
|  | 127042681 | Bz-Arg-Lys-D-nTyr-NH2 | 554.6 | C27H38N8O5 |
|  | 127042987 | Bz-Arg-Lys-nTyr(Bn)-NH2 | 644.8 | C34H44N8O5 |
|  | 127042988 | Bz-Arg-Lys-D-nTyr(Bn)-NH2 | 644.8 | C34H44N8O5 |
|  | 127043014 | 3-[5-(3-Hydroxyprop-1-ynyl)thiophen-2-yl]-4-methoxybenzoic acid | 288.3 | C15H12O4S |
|  | 127043015 | 5-[5-(3-Hydroxyprop-1-ynyl)thiophen-2-yl]-4-methoxy-2-methylbenzoic acid | 302.3 | C16H14O4S |
|  | 127043016 | 3-[5-[2-methoxy-5-(2H-tetrazol-5-yl)phenyl]thiophen-2-yl]prop-2-yn-1-ol | 312.3 | C15H12N4O2S |
|  | 127043018 | 3-[5-[5-(1H-imidazol-2-yl)-2-methoxy-4-methylphenyl]thiophen-2-yl]prop-2-yn-1-ol | 324.4 | C18H16N2O2S |
|  | 127043019 | N-[5-[5-(3-hydroxyprop-1-ynyl)thiophen-2-yl]-4-methoxy-2-methylphenyl]sulfonylacetamide | 379.5 | C17H17NO5S2 |
|  | 127043024 | N-(benzenesulfonyl)-5-[5-(3-hydroxyprop-1-ynyl)thiophen-2-yl]-4-methoxy-2-methylbenzamide | 441.5 | C22H19NO5S2 |
|  | 127043025 | N-(benzenesulfonyl)-5-[5-(3-hydroxyprop-1-ynyl)thiophen-2-yl]-2,4-dimethoxybenzamide | 457.5 | C22H19NO6S2 |
|  | 127043211 | 4-chloro-5-[5-(3-hydroxyprop-1-ynyl)thiophen-2-yl]-2-methoxy-N-(3-methoxyphenyl)sulfonylbenzamide | 492 | C22H18ClNO6S2 |
|  | 127043212 | N-[5-[5-(3-hydroxyprop-1-ynyl)thiophen-2-yl]-4-methoxy-2-methylphenyl]sulfonylquinoline-8-carboxamide | 492.6 | C25H20N2O5S2 |
|  | 127043361 | 2-[3-(Carboxymethyl)-5-(5-chlorothiophen-2-yl)phenyl]acetic acid | 310.8 | C14H11ClO4S |
|  | 127044830 | 2-[2-[(3-Chlorophenyl)methyl]-4,5-dihydroxyphenyl]-5,7-dihydroxychromen-4-one | 410.8 | C22H15ClO6 |
|  | 127044864 | 2-[3-[5-(3-Hydroxyprop-1-ynyl)thiophen-2-yl]-4-methoxyphenyl]acetic acid | 302.3 | C16H14O4S |
|  | 127045349 | 2-[3-(5-Bromothiophen-2-yl)-5-(carboxymethyl)phenyl]acetic acid | 355.21 | C14H11BrO4S |
|  | 134133625 | 4-[[3-[[2-Amino-4-(trifluoromethyl)phenyl]carbamoylamino]phenyl]sulfonylamino]benzoic acid | 494.4 | C21H17F3N4O5S |
|  | 134145727 | 4-[[4-[[2-Amino-4-(trifluoromethyl)phenyl]carbamoylamino]phenyl]sulfonylamino]benzoic acid | 494.4 | C21H17F3N4O5S |
|  | 134151968 | 4-[[4-[(2-Amino-4-chlorophenyl)carbamoylamino]phenyl]sulfonylamino]benzoic acid | 460.9 | C20H17ClN4O5S |
|  | 134154289 | 4-[4-[(2-Amino-4-chlorophenyl)carbamoylamino]phenyl]sulfonyloxybenzoic acid | 461.9 | C20H16ClN3O6S |
|  | 135427418 | 6-amino-2-[(2-{1-[(2H-1,3-benzodioxol-5-yl)methyl]-2,5-dimethyl-1H-pyrrol-3-yl}-2-oxoethyl)sulfanyl]-3,4-dihydropyrimidin-4-one | 412.5 | C20H20N4O4S |
|  | 135434165 | 3'-Deoxy-guanosine-5'-triphosphate | 507.18 | C10H16N5O13P3 |
|  | 135476298 | 4-Hydroxy-1-methyl-3-(5-thiophen-2-yl-4,5-dihydro-1,2-oxazol-3-yl)quinolin-2-one | 326.4 | C17H14N2O3S |
|  | 135555030 | N'~1~,N'~6~-bis[(1E)-(2,4-dihydroxyphenyl)methylene]hexanedihydrazide | 414.4 | C20H22N4O6 |
|  | 135976298 | 2-chloro-5-[(2Z)-2-[[4-(diethylamino)-2-hydroxyphenyl]methylidene]hydrazinyl]benzoic acid | 361.8 | C18H20ClN3O3 |
|  | 136205805 | ethyl 3-[(E)-(1H-benzimidazol-2-ylhydrazinylidene)methyl]-4-hydroxy-5-methylbenzoate | 338.4 | C18H18N4O3 |
|  | 136205806 | ethyl 3,5-bis[(E)-(1H-benzimidazol-2-ylhydrazinylidene)methyl]-4-hydroxybenzoate | 482.5 | C25H22N8O3 |
|  | 136205808 | ethyl 4-hydroxy-3,5-bis[(E)-[(4-nitrophenyl)hydrazinylidene]methyl]benzoate | 492.4 | C23H20N6O7 |
|  | 136205825 | ethyl 4-hydroxy-3,5-bis[(E)-[(4-propan-2-ylphenyl)hydrazinylidene]methyl]benzoate | 486.6 | C29H34N4O3 |
|  | 137243533 | 3-[5-[5-(3-hydroxyprop-1-ynyl)thiophen-2-yl]-4-methoxy-2-methylphenyl]-4H-1,2,4-oxadiazol-5-one | 342.4 | C17H14N2O4S |

**Fig S1.** Root mean square fluctuations (RMSF) for each residue of M^pro^ complexed with (a) **1**, (b) **2**, (c) **3**, (d) **4**, (e) **5**, and (f) control compound (X77).


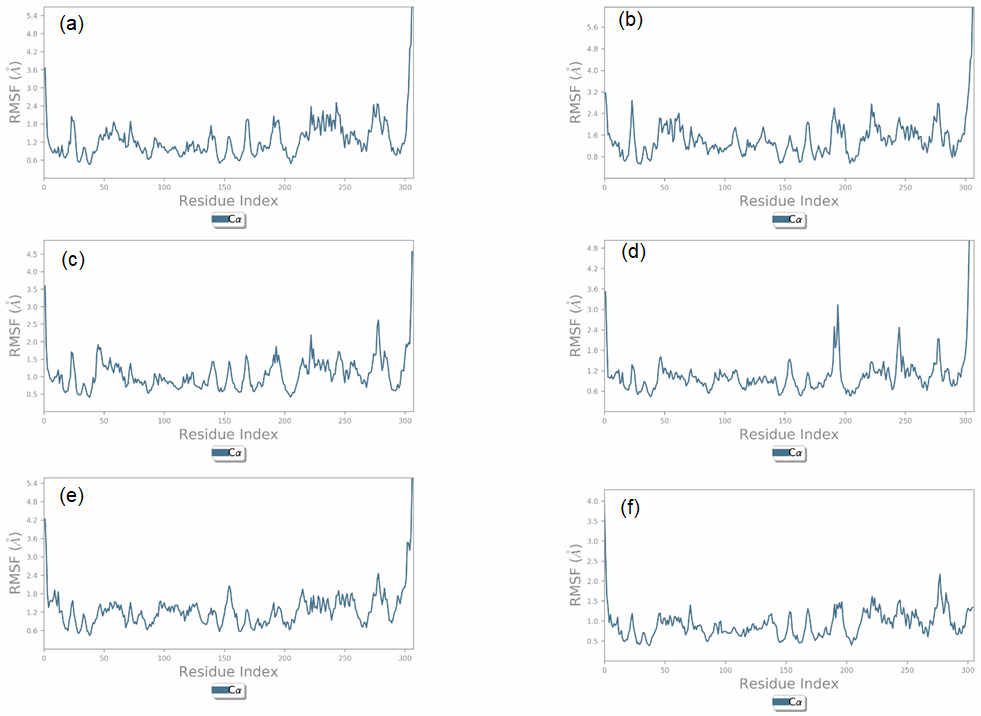


**Fig S2.** Proportion of various type of interactions contributed to total MM/GBSA shown by key residues in the binding site of M^pro^ with all five screened compounds with (a) **1**, (b) **2**, (c) **3**, (d) **4**, (e) **5**, and (f) control compound (X77).


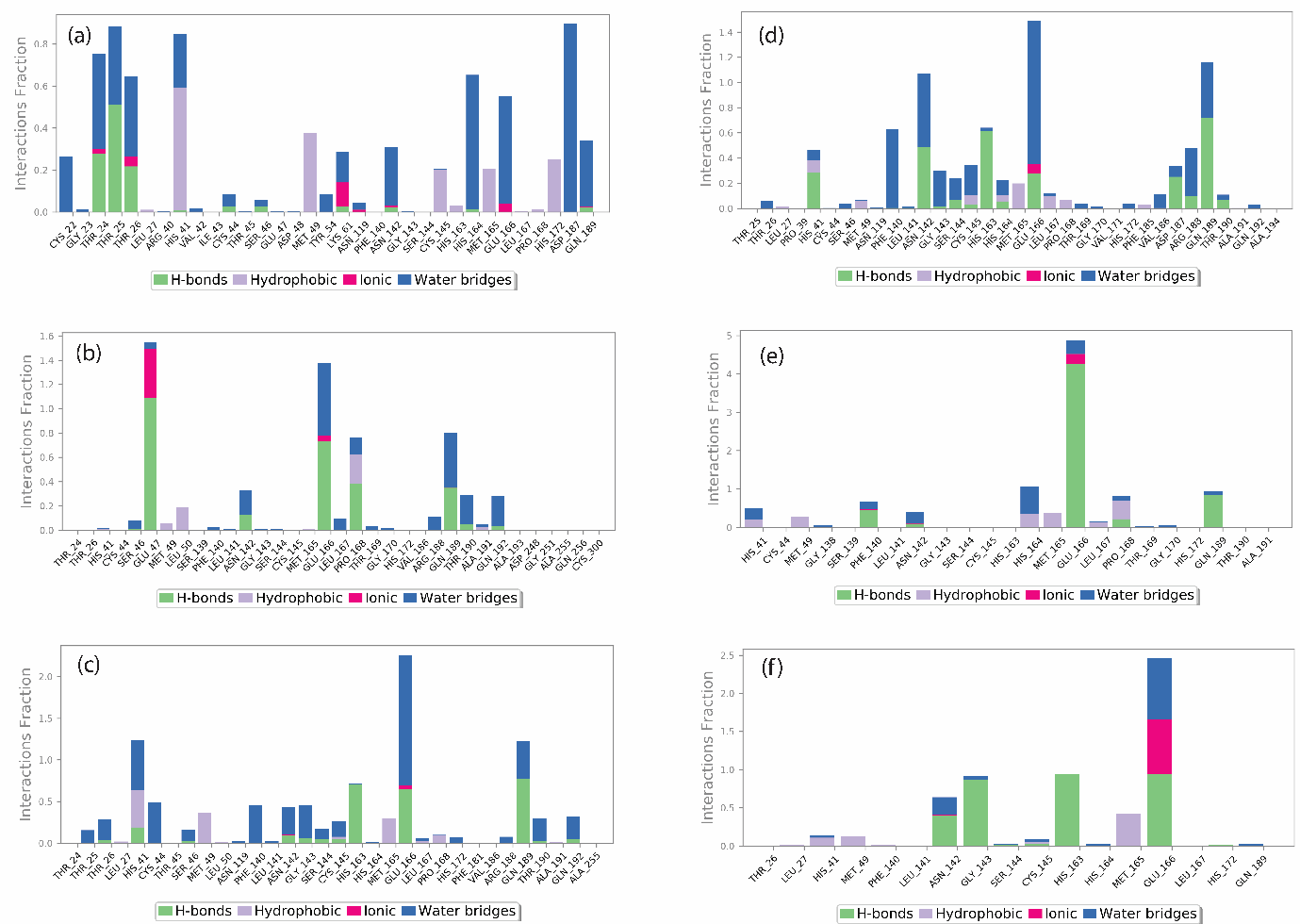


**Fig S3.**  Dynamic flexibility depiction using first and last pose obtained from MD simulation for (a) M^pro^-CHEMBL1940602 (b) M^pro^-CHEMBL2036486 (c) M^pro^-CHEMBL3628485 (d) M^pro^-CHEMBL200972 (e) M^pro^-CHEMBL2036488, and (f) M^pro^-X77 (control)


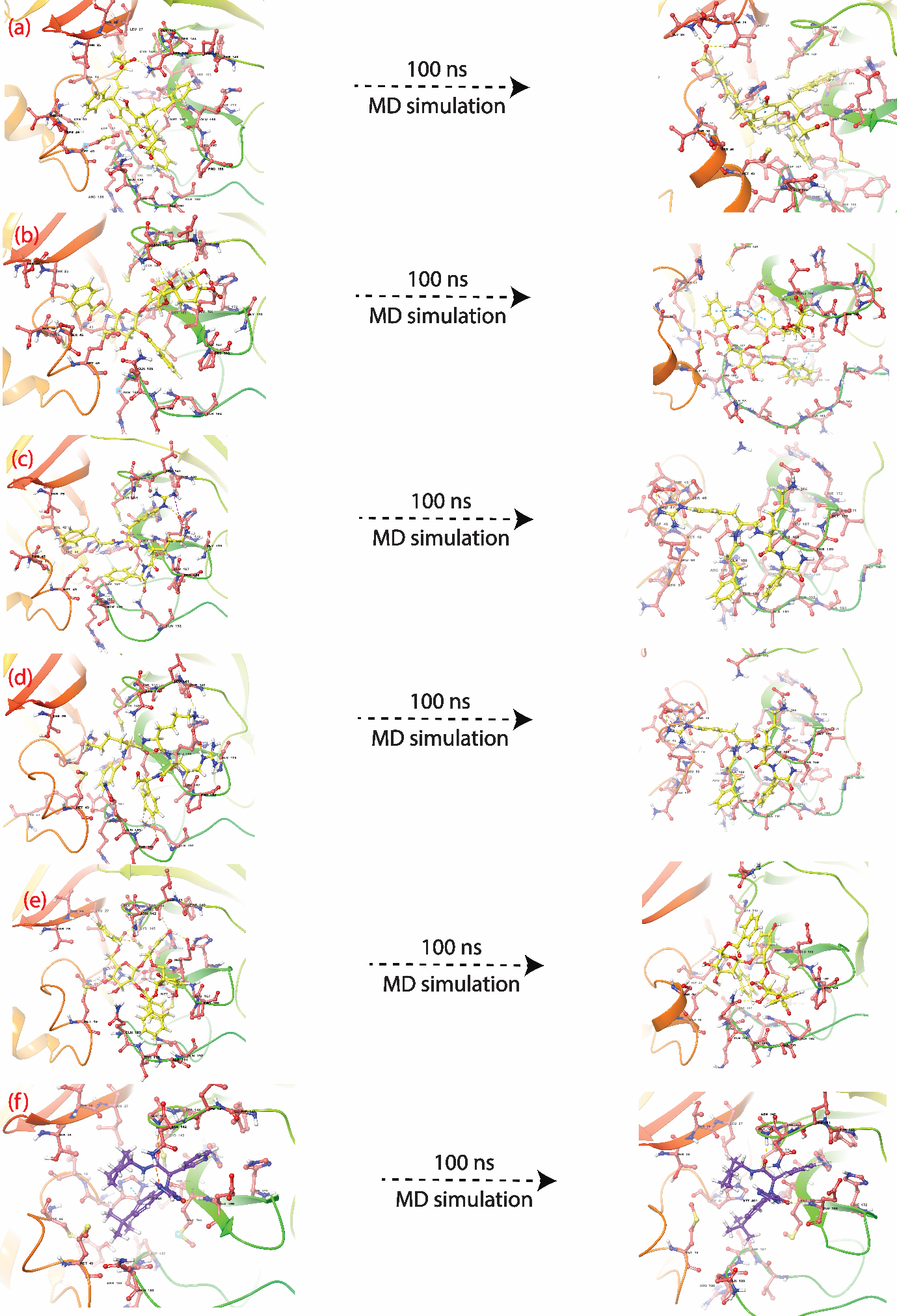

Supplement: S1 File — (DOCX) [file pone.0277328.s001.docx]
