# Supplementary material for: Repositioning of anti-dengue compounds against SARS-CoV-2 as viral polyprotein processing inhibitor
Source: PLoS One. 2022 Nov 16;17(11):e0277328. doi: 10.1371/journal.pone.0277328 (PMC9668197; doi:10.1371/journal.pone.0277328)

**Repositioning of Anti-Dengue Compounds Against SARS-CoV-2 as Viral Polyprotein Processing Inhibitor**

Leena H. Bajrai^1,2^, Arwa A. Faizo^1,3^, Areej A. Alkhaldy^1,4^, Vivek Dhar Dwivedi^5*^, and Esam I. Azhar^1,3*^

^1^Special Infectious Agents Unit – BSL3, King Fahd Medical Research Center, King Abdulaziz University, Jeddah 21362, Saudi Arabia

^2^Biochemistry Department, Faculty of Sciences, King Abdulaziz University, Jeddah 21362, Saudi Arabia

^3^Department of Medical Laboratory Sciences, Faculty of Applied Medical Sciences, King Abdulaziz University, Jeddah 21362, Saudi Arabia

^4^Clinical Nutrition Department, Faculty of Applied Medical Sciences, King Abdulaziz University, Jeddah, 21589, Saudi Arabia

^5^Center for Bioinformatics, Computational and Systems Biology, Pathfinder Research and Training Foundation, Greater Noida 201308, India.

^6^Bioinformatics Research Division, Quanta Calculus, Greater Noida, 201310, India

***Corresponding authors**

EIA; Email: [eazhar@kau.edu.sa](mailto:eazhar@kau.edu.sa)

VDD; Email: [vivek_bioinformatics@yahoo.com](mailto:vivek_bioinformatics@yahoo.com); [vivekdhardwivedi@quantacalculus.com](mailto:vivekdhardwivedi@quantacalculus.com)


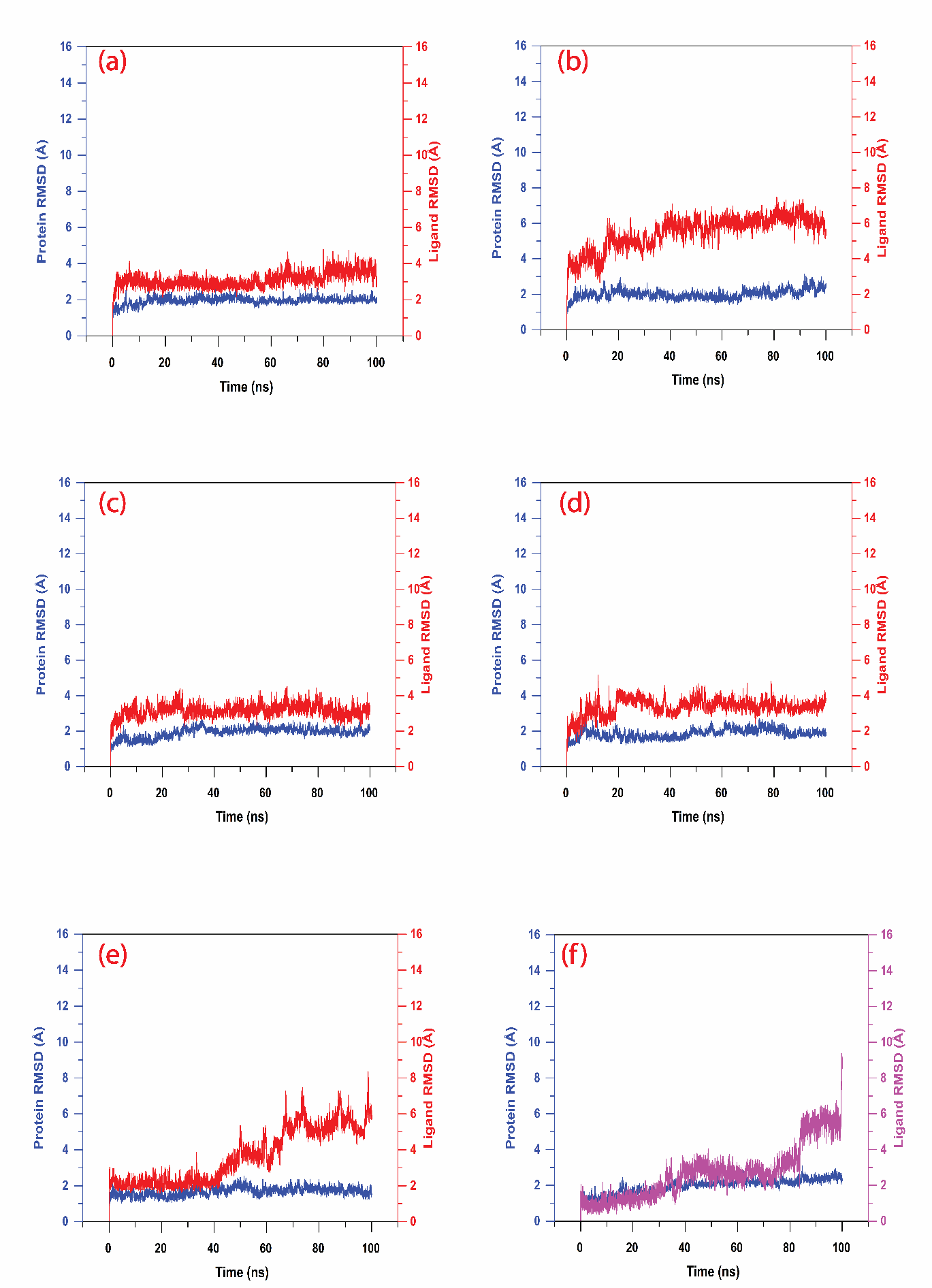
**Figure-B1**. Root mean square deviation (RMSD) plots for (a) Mpro -CHEMBL1940602 (b) Mpro 367 -CHEMBL2036486 (c) Mpro -CHEMBL3628485 (d) Mpro -CHEMBL200972 (e) Mpro -CHEMBL2036488, and (fCH) Mpro 368 -X77 (control), 369 calculated over the period of 100 ns MD simulation.


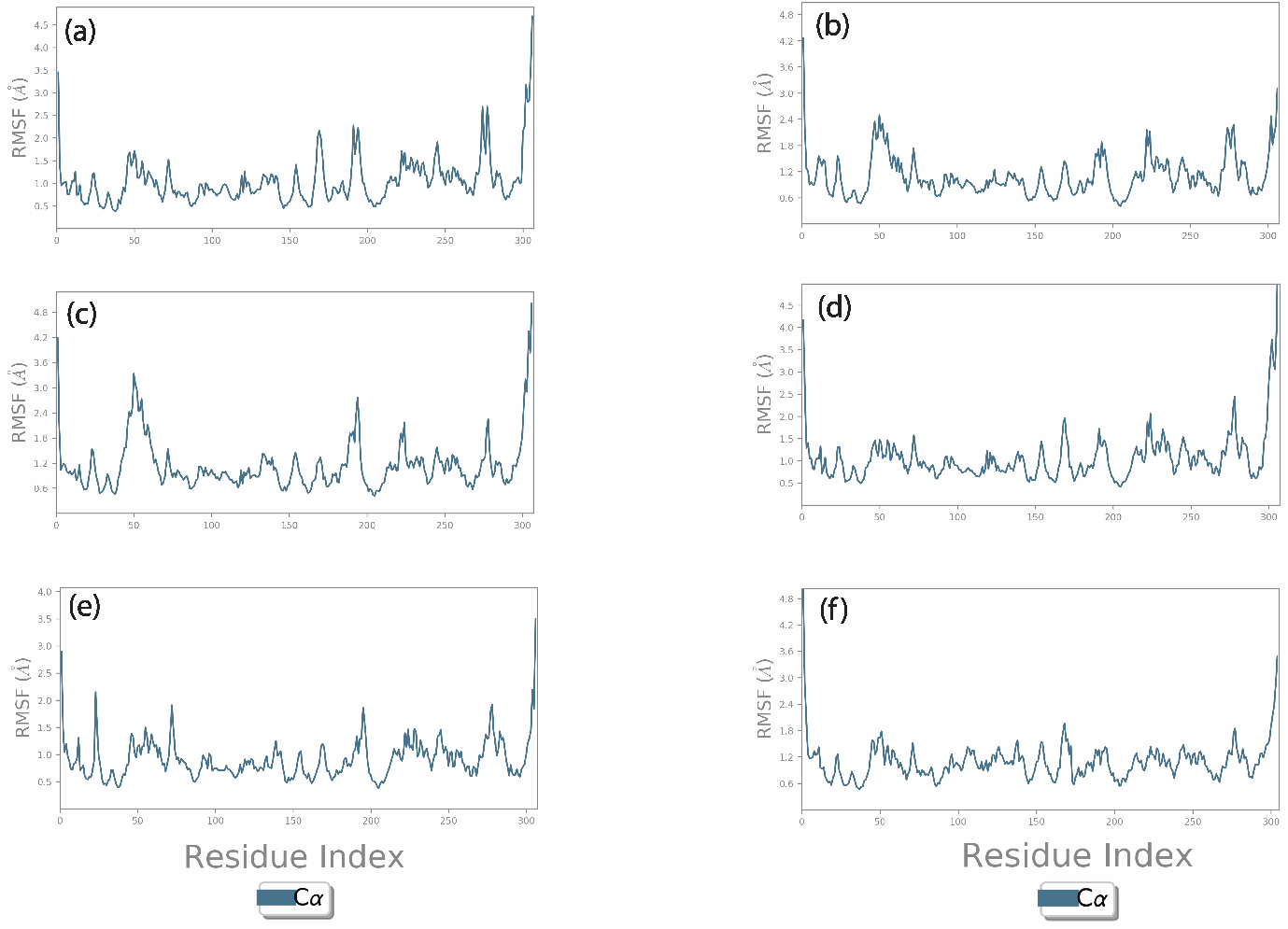


**Figure-B2.** Root mean square fluctuation (RMSF) plots of protein for (a) Mpro -CHEMBL1940602 (b) Mpro 367 -CHEMBL2036486 (c) Mpro -CHEMBL3628485 (d) Mpro -CHEMBL200972 (e) Mpro -CHEMBL2036488, and (fCH) Mpro 368 -X77 (control), 369 calculated over the period of 100 ns MD simulation.


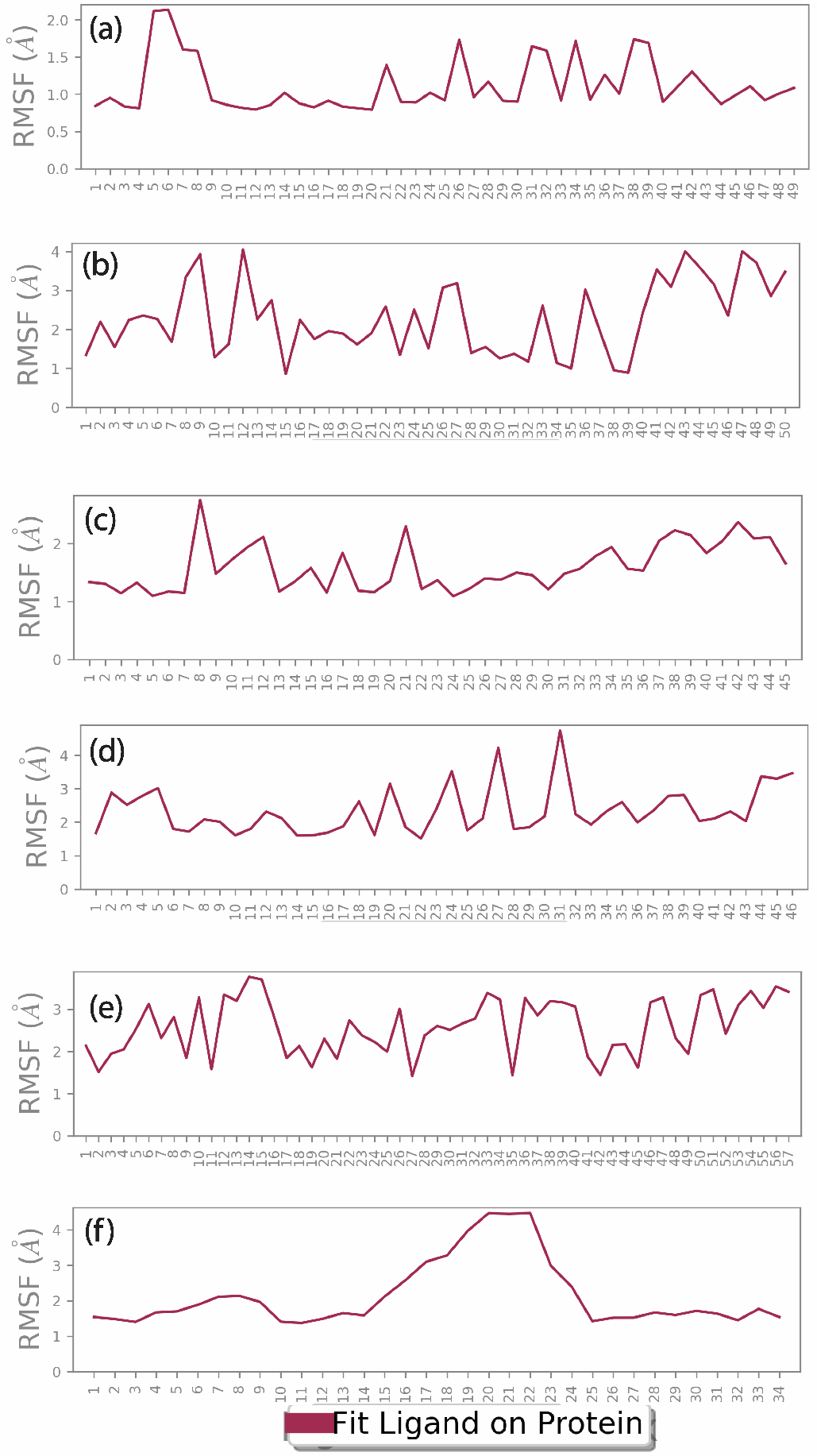


**Figure-B3**. Root mean square fluctuation (RMSF) plots of ligands for (a) Mpro -CHEMBL1940602 (b) Mpro 367 -CHEMBL2036486 (c) Mpro -CHEMBL3628485 (d) Mpro -CHEMBL200972 (e) Mpro -CHEMBL2036488, and (fCH) Mpro 368 -X77 (control), 369 calculated over the period of 100 ns MD simulation.


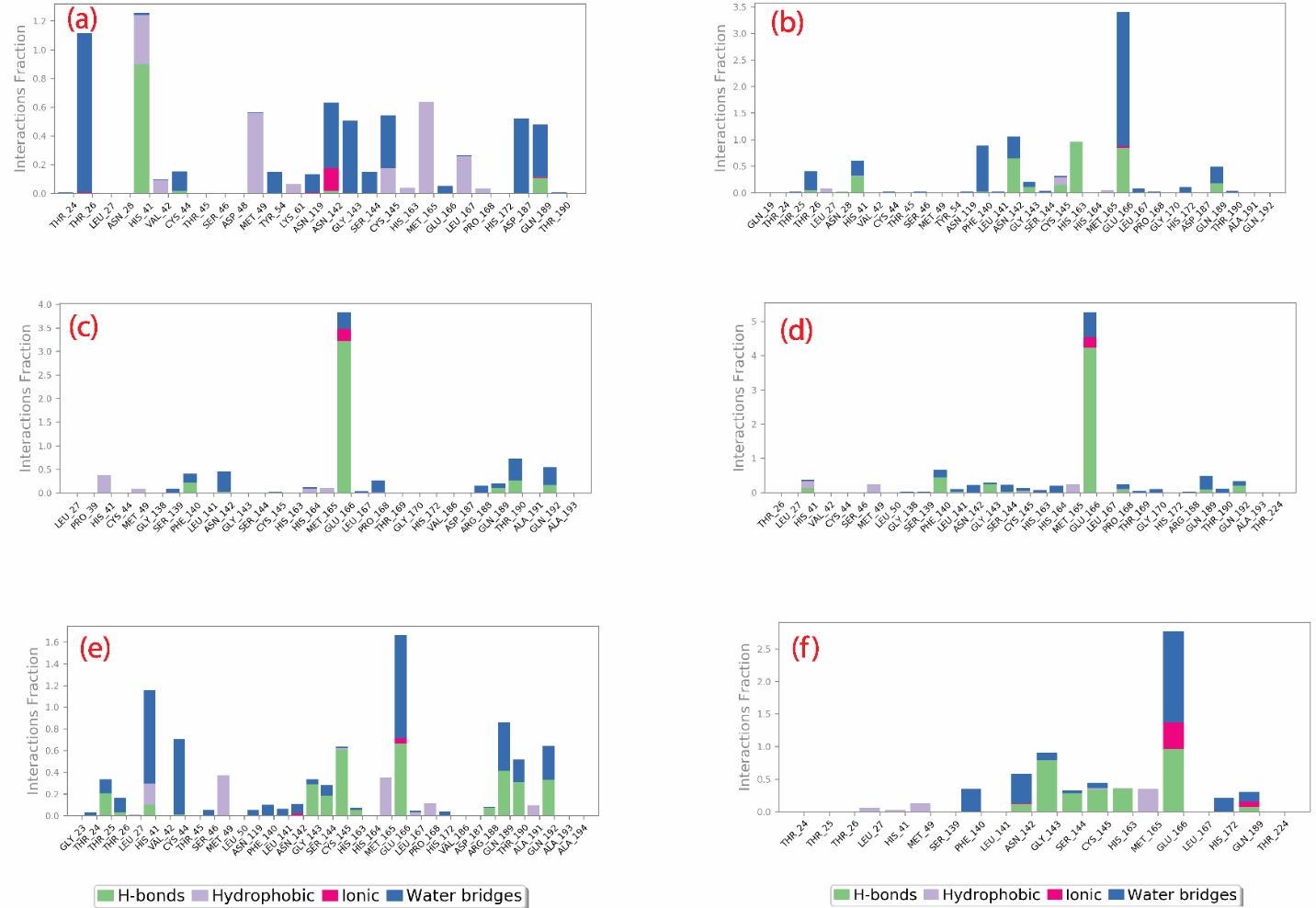


**Figure-B4.** Protein-ligand interaction mapping for (a) Mpro -CHEMBL1940602 (b) Mpro 367 -CHEMBL2036486 (c) Mpro -CHEMBL3628485 (d) Mpro -CHEMBL200972 (e) Mpro -CHEMBL2036488, and (fCH) Mpro 368 -X77 (control), 369 calculated over the period of 100 ns MD simulation.

**Figure-B5.** Protein-ligand conact for (a) Mpro -CHEMBL1940602 (b) Mpro 367 -CHEMBL2036486 (c) Mpro -CHEMBL3628485 (d) Mpro -CHEMBL200972 (e) Mpro -CHEMBL2036488, and (fCH) Mpro 368 -X77 (control), 369 calculated over the period of 100 ns MD simulation.


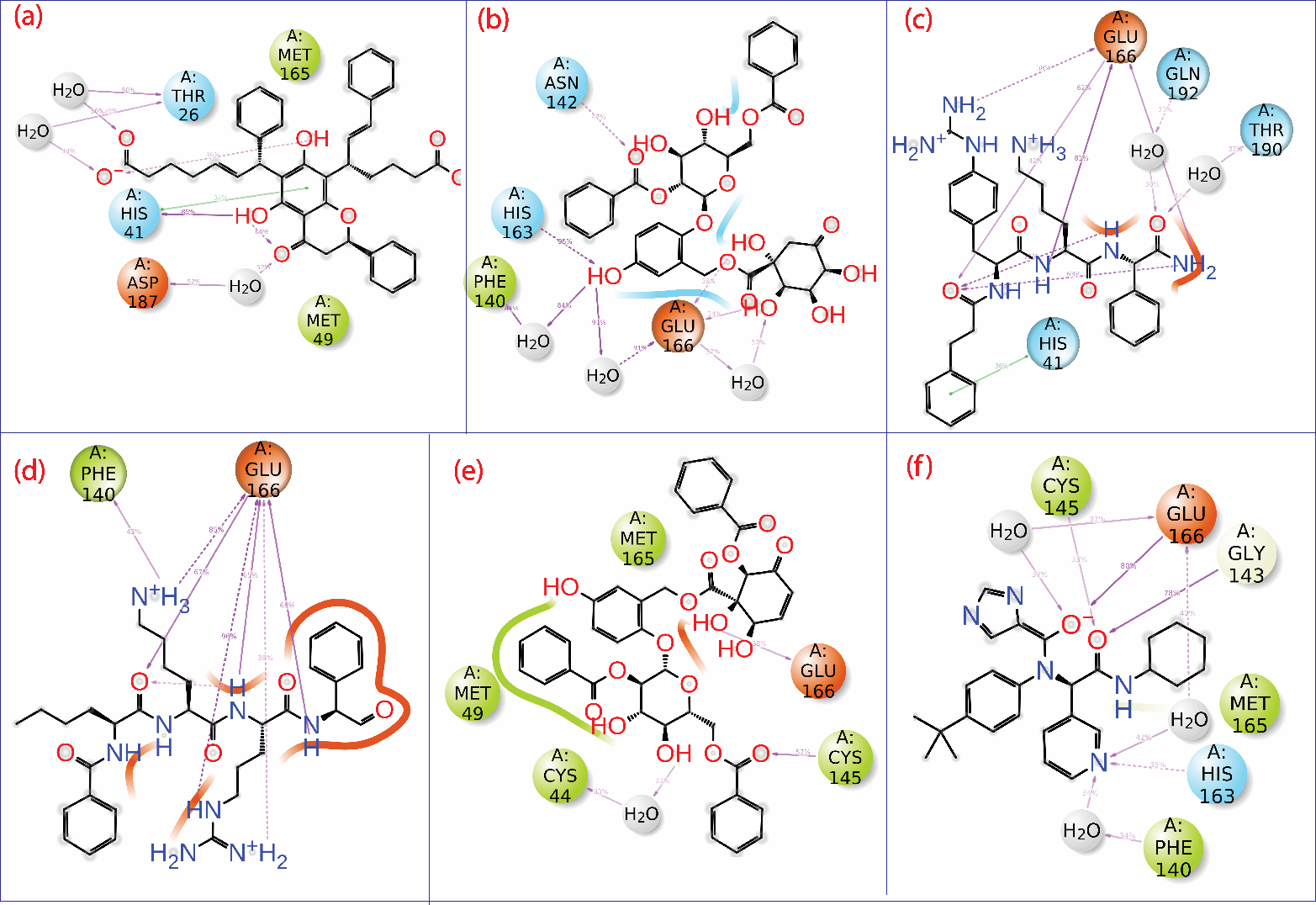

Supplement: S2 File — (DOCX) [file pone.0277328.s002.docx]
